# Molecular Pharmacology of VEGF-A Isoforms: Binding and Signalling at VEGFR2

**DOI:** 10.3390/ijms19041264

**Published:** 2018-04-23

**Authors:** Chloe J. Peach, Viviane W. Mignone, Maria Augusta Arruda, Diana C. Alcobia, Stephen J. Hill, Laura E. Kilpatrick, Jeanette Woolard

**Affiliations:** 1 Division of Physiology, Pharmacology and Neuroscience, School of Life Sciences, Queen’s Medical Centre, University of Nottingham, Nottingham NG7 2UH, UK; chloe.peach@nottingham.ac.uk (C.J.P.); viviane.mignone@nottingham.ac.uk (V.W.M.); maria.arruda@nottingham.ac.uk (M.A.A.); mbxdc1@nottingham.ac.uk (D.C.A.); steve.hill@nottingham.ac.uk (S.J.H.); 2 Centre of Membrane Proteins and Receptors (COMPARE), University of Birmingham and University of Nottingham, Midlands NG7 2UH, UK; 3 CAPES-University of Nottingham Programme in Drug Discovery, Queen’s Medical Centre, University of Nottingham, Nottingham NG7 2UH, UK

**Keywords:** angiogenesis, endothelial cells, blood vessel, splicing, receptor tyrosine kinase inhibitors

## Abstract

Vascular endothelial growth factor-A (VEGF-A) is a key mediator of angiogenesis, signalling via the class IV tyrosine kinase receptor family of VEGF Receptors (VEGFRs). Although VEGF-A ligands bind to both VEGFR1 and VEGFR2, they primarily signal via VEGFR2 leading to endothelial cell proliferation, survival, migration and vascular permeability. Distinct VEGF-A isoforms result from alternative splicing of the *Vegfa* gene at exon 8, resulting in VEGF_xxx_a or VEGF_xxx_b isoforms. Alternative splicing events at exons 5–7, in addition to recently identified posttranslational read-through events, produce VEGF-A isoforms that differ in their bioavailability and interaction with the co-receptor Neuropilin-1. This review explores the molecular pharmacology of VEGF-A isoforms at VEGFR2 in respect to ligand binding and downstream signalling. To understand how VEGF-A isoforms have distinct signalling despite similar affinities for VEGFR2, this review re-evaluates the typical classification of these isoforms relative to the prototypical, “pro-angiogenic” VEGF_165_a. We also examine the molecular mechanisms underpinning the regulation of VEGF-A isoform signalling and the importance of interactions with other membrane and extracellular matrix proteins. As approved therapeutics targeting the VEGF-A/VEGFR signalling axis largely lack long-term efficacy, understanding these isoform-specific mechanisms could aid future drug discovery efforts targeting VEGF receptor pharmacology.

## 1. Introduction

Angiogenesis is the formation of new blood vessels by the sprouting of endothelial cells from pre-existing vasculature [1]. While vasculogenesis (formation of angioblast-derived blood vessels) occurs mainly during embryonic development, angiogenesis takes place throughout adult life [2,3], playing a vital role in physiological events such as wound repair [4], the oestrous cycle and placentation [5]. In addition to its importance in normal physiology, angiogenesis is also a key feature associated not only with cancer [6], but also with several pathological conditions including age-related macular degeneration [7], rheumatoid arthritis [8], psoriasis [9], diabetes-induced ocular neovascularisation [10], inflammatory diseases [11], ischaemia/reperfusion injury [12], infantile haemangioma and atherosclerosis [13,14]. Despite their distinct aetiologies, these disorders can be characterised as angiogenesis-dependent diseases [15] and are either caused or exacerbated by an imbalance between the production/activity of anti- and pro-angiogenic factors [16]. Among the pro-angiogenic endogenous molecules, vascular endothelial growth factor (VEGF) is a major regulator of blood vessel formation in health and disease [1]. Initially, VEGF was termed vascular permeability factor (VPF) due to its ability to increase the permeability of blood vessels [17,18]. Following subsequent observations of the additional effects of VPF on endothelial cells and concomitant cloning, VPF was renamed VEGF [19].

The VEGF family of proteins comprises VEGF-A, VEGF-B [20], VEGF-C, VEGF-D, Placental Growth Factor (PlGF) [21], the virus-encoded VEGF-E and the snake venom-derived VEGF-F [22,23,24]. VEGF-A is the best characterised family member being the most potent stimulator of angiogenic processes and therefore a target of numerous anti-cancer therapeutics [25]. VEGF-A is a large anti-parallel homodimeric peptide that belongs to the “Cys-loop” superfamily of proteins, based on a central knot motif of cysteine residues that form intramolecular disulphide bonds when assembled into a folded structure [24,26]. VEGF-A is secreted by many cell types such as endothelial cells [27,28], fibroblasts [29], smooth muscle cells [30], platelets [31], neutrophils [32], macrophages and approximately 60% of all tumours [33]. VEGF-A secretion is also induced by ischemia and inflammatory stimuli [34]. Cellular responses to VEGF-A are mainly driven by their binding to their cognate receptor—the vascular endothelial growth factor receptors (VEGFRs). VEGFRs belong to the class IV receptor tyrosine kinase (RTK) family [35] and show similarities to type III RTKs platelet derived growth factor receptor (PDGFR), macrophage colony stimulating factor receptor (M-CSFR), c-KIT and fms-like tyrosine kinase 3 (FLT3) [36]. There are three VEGFR subtypes which are encoded by separate genes: VEGFR1 (Flt-1 in mice) and VEGFR2 (Flk-1; KDR) are structurally similar, whereas VEGFR3 (Flt-4) has a proteolytically processed extracellular domain [37,38]. VEGFRs are expressed by endothelial cells, macrophages, hematopoietic cells and smooth muscle cells [39,40,41]. Signalling of VEGF-A isoforms via VEGFR1 and VEGFR2 drive physiological and pathophysiological angiogenesis, whereas lymphangiogenesis is mediated by VEGF-C/D isoforms via VEGFR3 [42]. Although VEGFR1 has a higher affinity for VEGF-A than VEGFR2, it shows decreased tyrosine kinase activity and is therefore largely considered a decoy receptor that can negatively modulate VEGFR2 activity [43,44]. While VEGFR1 also plays a role in immune cell differentiation [45,46], VEGFR1 is beyond the scope of this review (reviewed in [47]).

Alternative splicing of the *Vegfa* gene leads to different VEGF-A isoforms which have been proposed to promote distinct signalling outcomes [16]. Through quantifying and comparing the pharmacology of VEGF-A isoforms at VEGFR2, we can begin to comprehend how they differ as distinct endogenous ligands. This could ultimately enable better understanding of molecular mechanisms that give rise to distinct physiological outcomes with relevance in health and disease [16] and future drug discovery efforts [25]. In this review, we have explored in detail the molecular pharmacology of VEGF-A isoforms in terms of their receptor binding to VEGFR2 and downstream signalling with particular reference to the influence of agonist efficacy and signalling coupling on physiological outcomes.

## 2. Generation of VEGF-A Isoforms by Alternative Splicing

**Figure 1:**
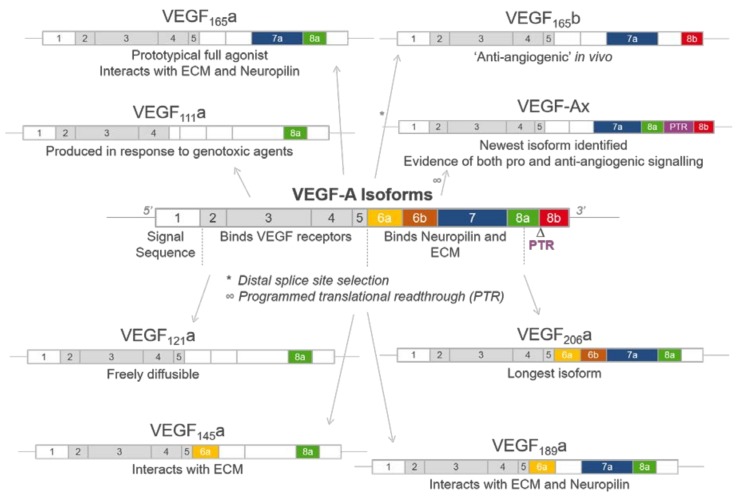
Schematic illustrating the structure of vascular endothelial growth factor A (VEGF-A) isoforms. The VEGF-A gene consists of eight exons, which can be alternatively spliced to generate a range of VEGF-A isoforms. These isoforms differ in length and have been designated VEGF_xxx_, where xxx represents the number of amino acids present. Each exon contains residues identified as conferring distinct properties if included in the resultant isoform, including VEGFR2, extracellular matrix (ECM) and Neuropilin (NRP) binding. A major site of alternative splicing occurs at exon 8, whereby proximal splicing results in the prototypical VEGF_xxx_a forms and distal splicing the “anti-angiogenic” VEGF_xxx_b isoforms containing exon 8b. Additionally, post translational read-through (PTR) using a non-canonical stop codon results in the VEGF-Ax isoform which contains a 22 amino acid extension in its C terminal domain.

The regulation of vascular supply relies on tight regulation between factors that promote (pro-angiogenic) or inhibit (anti-angiogenic) vessel development, via a mechanism that is reactive to changes in oxygen and nutrient levels. VEGF-A transcription is affected by the local cellular environment, such as during hypoxia [48,49] following secretion of growth factors, cytokines and hormones, shear stress, genotoxic agents [50] and the activity of both oncogenes and tumour suppressor genes [51]. The human *Vegf*a gene is located on chromosome 6p21.1 [52], with a coding region spanning approximately 14 kilobases consisting of eight exons and seven introns. Alternative splicing of this pre-mRNA selectively removes intron regions and joins specific combinations of exons to generate distinct VEGF-A isoforms [53] (Figure 1). Alternative splicing is advantageous in expanding the repertoire of possible VEGF-A isoforms that can be produced from a single gene [54]. These isoforms differ in respect to their length and are designated VEGF_xxx_, where xxx represents the number of amino acids present in the final protein sequence. To date 16 distinct VEGFA isoforms have been identified most commonly from six transcripts: VEGF_111_, VEGF_121_, VEGF_145_, VEGF_165_, VEGF_189_, and VEGF_206_ [16,55,56]. An additional isoform, VEGF-Ax, was also identified in 2014 that arises from programmed translational read-through (PTR) [56]. VEGF_165_a was the first isoform characterised and remains the most extensively investigated in respect to its function, signalling, expression and pathological roles [19]. As a potent stimulator of angiogenesis, VEGF_165_a is considered the prototypical pro-angiogenic VEGF-A isoform. Altered VEGF-A isoform expression has been well documented in tissues during physiological and/or pathological conditions [57,58,59,60,61].

Boundaries between exons are defined by splicing sites which are recognised by a dynamic complex of proteins located in the nucleus called the spliceosome [62], containing five small nuclear ribonucleoproteins (snRNPs)—U1, U2, U4, U5 and U6—plus associated accessory proteins U2AF and SF1. VEGF-A splicing is also regulated by a series of RNA binding proteins, most commonly the serine/arginine (SR) proteins, chiefly SRSF1, SRSF2, SRSF5 and SRSF6 [54]. SR proteins are phosphorylated in the cytoplasm at multiple serine/arginine and proline/serine repeats to enable their subsequent translocation to the nucleus and allows a degree of spatial regulation of splicing. Once in the nucleus, SR proteins typically bind to regulatory sites in VEGF-A pre-mRNA—exonic sequence enhancers [63,64]—which trigger exon removal. One such kinase responsible for phosphorylating SR proteins is the constitutively active kinase SRPK1 [63]. Alterations in SRPK1 expression have been identified (via both upregulation and downregulation) in a range of malignancies. This has led to the development of SRPK1 inhibitors targeting aberrant angiogenesis through altering splicing of endogenous VEGF-A isoforms [63,65,66].

**Figure 2:**
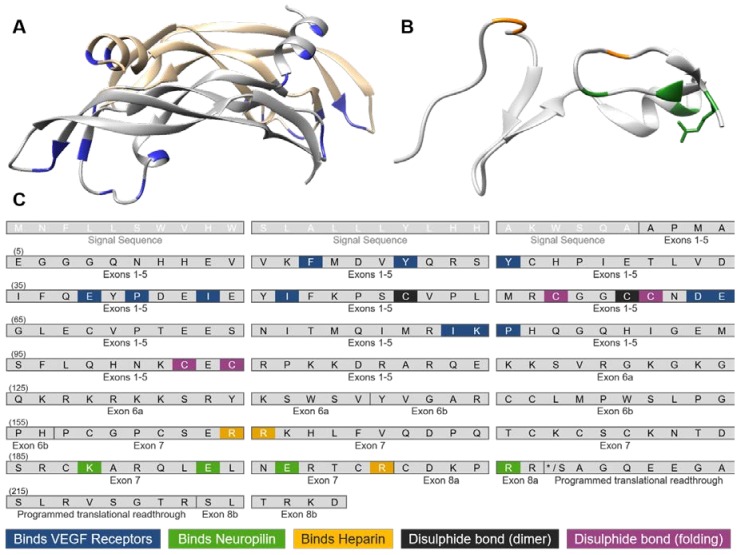
Molecular structure of VEGF-A. (**A**) Anti-parallel homodimeric structure of VEGF-A encoded by exons 2–5 (PDB:1VPF), showing distinct VEGF monomers in grey and gold and residues interacting with VEGF receptors shown in blue; (**B**) C-terminus of VEGF_165_a is encoded by exons 7–8a (PDB:4DEQ), with residues that bind heparin (yellow) and Neuropilin-1 (green) highlighted; (**C**) Amino acid residues present in exons of the human VEGF-A sequence that interact with known binding partners. The open reading frame was derived from transcript NM_001025366.2 with exons denoted according to UniProt (P15692) and residues numbered according to residues in the final VEGF-A peptide following cleavage of the signal sequence. Based on published X-ray crystal structures, residues are highlighted that form non-covalent interactions with VEGFR1 [88], VEGFR2 [84], Neuropilin-1 [89] or heparin [70]. Cysteine residues forming intermolecular or intramolecular disulphide bonds, important for dimeric or folding structure, respectively, are also highlighted [26].

The distinct exons included in each isoform confer different properties (Figure 1 and Figure 2). Exons 1–5 are constitutive exons and are therefore present in all VEGF-A isoforms. These encode a signal sequence (exons 1/2) that is cleaved in the processed form of VEGF, a glycosylation site (Asp74), a potential plasmin cleavage site (Arg110 and Ala111) [67] and residues responsible for VEGFR1 and VEGFR2 binding (Figure 2A,C) [16,68]. A major site of alternative splicing of the *Vegf*a gene centres on exons 6 and 7. Residues in exons 6a and 7 interact with electronegative heparin sulphate in the extracellular matrix, which has important implications for isoform bioavailability [69,70,71]. The shorter isoforms VEGF_111_ and VEGF_121_ both lack exons 6 and 7, and as a consequence are not tethered to the extracellular matrix (ECM) and are freely diffusible [70,72]. In contrast the longer isoforms VEGF_145_, VEGF_189_ and VEGF_206_ containing both exons 6a and 7 can bind with high affinity to heparin sulphate glycoproteins [73] (Figure 1). The prototypical VEGF_165_a is an intermediate between these freely diffusible and bound isoforms, in that following secretion 50–70% remains cell or ECM bound [72].

A second major site of alternative splicing is driven by the choice of differential 3′ splice acceptor sites within exon 8. In 2002, Bates et al. identified the VEGF_xxx_b family of isoforms [74]. These isoforms arise due to distal splicing at a site located 66 base pairs downstream of the proximal splicing site, resulting in isoforms that contain exon 8b (Figure 1 and Figure 2) [51]. In respect to their sequences, VEGF_xxx_a and VEGF_xxx_b isoforms only differ in the six amino acids found at their C termini; VEGF_xxx_a isoforms end in the sequence CDKPRR, whereas VEGF_xxx_b isoforms terminate in SLTRKD [75]. Based on both in vitro and in vivo experimental evidence, VEGF_xxx_a isoforms are considered to be “pro-angiogenic” as major mediators of vascular permeability, cell proliferation, survival and migration, and angiogenesis [76]; in contrast, VEGF_xxx_b isoforms have been reported to have “anti-angiogenic” properties [74,77,78], with evidence that these isoforms may act as regulators and inhibitors of VEGF_xxx_a-induced pro-angiogenic activity [51,77]. Interestingly, in quiescent vessels, a higher proportion of total VEGF-A is represented by VEGF_165_b, which is then downregulated in cancer where a switch to pro-angiogenic isoform expression is observed to drive tumour angiogenesis [74,77,79]. Proximal or distal splicing of exon 8 can be influenced by external stimuli, as proximal splicing has been promoted by insulin like growth factor (IGF1) or tumour necrosis factor alpha (TNFα), whereas stimulation with tumour growth factor beta 1 (TGF-β1) has promoted distal splicing [51]. This bias was governed by the specific SR protein splice factor that was bound to a sequence within exon 8a (SRSF1) or 8b (SRSF6). It is worth noting that this bias may not be consistent in all cell types, however this highlights how isoform expression can be context dependent. There has been some debate as to the existence of VEGF_xxx_b isoforms physiologically [80,81], with genome wide RNA sequencing data of the human transcriptome questioning whether the relevant exon-exon junctions in the *Vegfa* gene are present [81].

VEGF-Ax, recently identified by Eswarappa et al. [56], is the result of extended translation beyond the canonical stop codon of VEGF-A mRNA due to the presence of an alternative stop codon within the 3′ untranslated region (Figure 1 and Figure 2). PTR is at least partially regulated by the A2/B1 ribonucleoprotein acting as a *trans* regulatory factor. The resultant VEGF-Ax therefore contains a 22 amino acid extension encompassing both exons 8a encoded CDKPRR and exon 8b encoded SLTRKD sequences [56,82]. The physiological role of VEGF-Ax is still yet to be fully elucidated with evidence it exhibits both “anti” and “pro-angiogenic” signalling [56,82].

## 3. VEGF-A Ligand/Receptor Binding

VEGFR2 is a large 151 kDa membrane protein consisting of 7 extracellular immunoglobulin (Ig)-like domains, a single transmembrane helix and a split intracellular kinase domain [83]. VEGF-A is an endogenous agonist for VEGFR2, binding the orthosteric ligand binding site across Ig-like domains 2 (D2) and D3 with a stoichiometry of one VEGF-A dimer across a VEGFR dimer [84,85]. X-ray and NMR structures have identified binding interfaces between VEGF-A and its receptors, confirming key exposed residues at each pole of the homodimer interacting with VEGFR1 [86,87,88] and VEGFR2 [84]. As each VEGF-A isoform contains residues encoded by exons 2–5 (Figure 1), residues interacting with VEGFR1 and VEGFR2 are not removed by alternative splicing (Figure 2A,C). Every isoform also contains cysteine residues that form intermolecular disulphide bonds such that all isoforms are dimeric, as well as forming intramolecular disulphide bonds assembling the Cys-loop folded structure (Figure 2A,C). In contrast, residues identified by structural studies that interact with co-receptor Neuropilin-1 (NRP1) or components of the ECM are absent in some VEGF-A isoforms (Figure 2B,C). The inability to crystallise both the N-terminal (exons 2–5) and C-terminal (exons 6–8) together suggest flexibility between these N- and C-terminal regions of VEGF-A, however the current lack of structural information on full-length VEGF-A isoforms has prevented understanding of the stoichiometry of macromolecular complex assembly.

**Figure 3:**
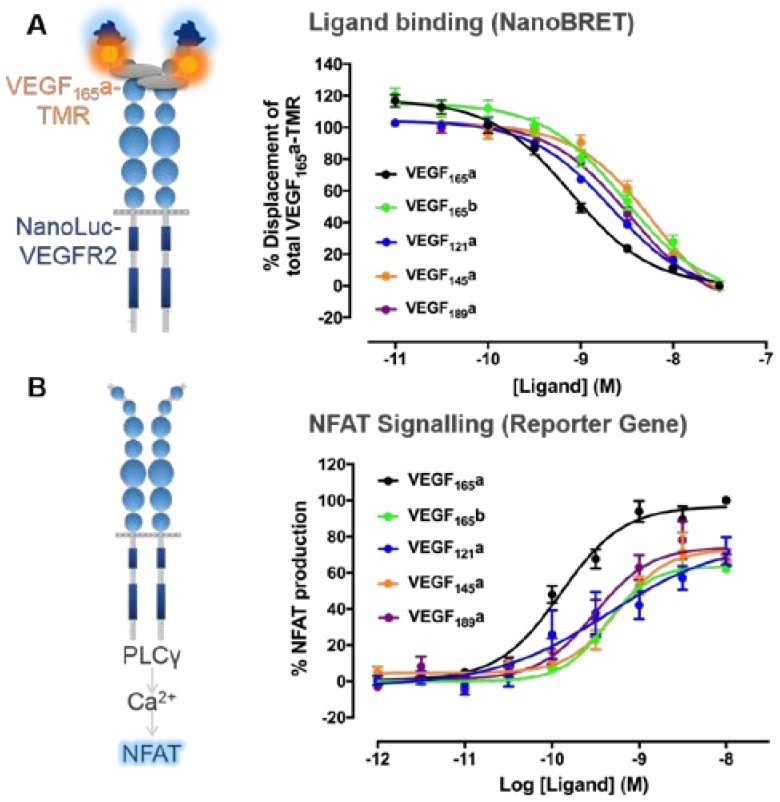
Quantifying VEGF-A isoform binding and downstream nuclear factor of activated T-cells (NFAT) signalling to derive pharmacological parameters. (**A**) Ligand binding affinities to VEGFR2 were quantified using HEK293 cells stably transfected with the full-length human VEGFR2 tagged at its *N*-terminus with the novel luciferase NanoLuc. Bioluminescence resonance energy transfer (BRET) experiments were then performed, whereby the close proximity of the donor NanoLuc tag with bound VEGF_165_a fluorescently with tetramethylrhodamine (VEGF_165_a-TMR) facilitates the non-radiative transfer of this energy to excite the acceptor TMR fluorophore which itself emits light at a longer wavelength. Cells were co-stimulated using a fixed concentration (3 nM) of single-site fluorescently labelled VEGF_165_a (VEGF_165_a-TMR) and increasing concentrations of competing unlabelled VEGF-A isoforms (60 min at 37 °C). These data were normalised to percentage displacement of VEGF_165_a-TMR alone and binding affinities (pK_i_ values) of unlabelled isoforms estimated using the Cheng–Prusoff equation with VEGF_165_a-TMR K_d_ values calculated from previous saturation experiments (see [97] for more details). (**B**) Functional potencies of VEGF-A isoforms were derived from an NFAT reporter gene assay, whereby a Firefly luciferase inserted downstream of the NFAT promoter sequence was used to investigate the potency of unlabelled VEGF-A isoforms in respect to stimulating downstream NFAT production. HEK293 cells stably expressing full-length human VEGFR2 were stimulated with a concentration response course of unlabelled VEGF-A isoforms (5 h at 37 °C/5% CO_2_). A luminescence readout was indicative of NFAT production. All responses were expressed as a percentage of 10 nM VEGF_165_a. The potency and efficacy of VEGF-A isoforms in respect to NFAT production were calculated using non-linear least square regression. All data were pooled from 4/5 independent experiments and expressed as ± S.E.M. Figures modified from Kilpatrick et al. (2017) [97].

Ligand binding affinity is the strength of the interaction between a ligand and its receptor, which can be quantified as the concentration of ligand required to bind 50% receptors at equilibrium (equilibrium dissociation constant, K_d_) [90]. Traditionally, VEGF-A binding has been investigated in cells expressing VEGFR2 using radiolabelled [^125^I]-VEGF_165_a (Table 1), in which radioligand affinity (K_d_) was determined by quantifying bound ligand with saturating concentrations [91,92]. Competing “hot” [^125^I]-VEGF_165_a with increasing concentrations of “cold” ligand allows the determination of affinity of unlabelled VEGF-A isoforms [44,70,77,93] (Table 1). Biochemical techniques have also been used to quantify VEGF-A binding affinities as a cell-free alternative using isolated receptors, including surface plasmon resonance (SPR) [85], solid-phase enzyme-linked assays [56,94] and thermodynamic calorimetry measurements [84,85]. Binding affinities determined using biochemical techniques using truncated VEGFR2 yielded higher estimated binding affinities than those determined with radioligand binding (Table 1), however radioligand binding experiments also have caveats beyond safety and cost. Recently, bioluminescence resonance energy transfer (BRET) was developed as a proximity-based technique using the novel luciferase NanoLuc to monitor ligand binding to GPCRs [95,96]. This allows ligand/receptor interactions to be monitored in real-time to receptors expressed within their native membrane environment. This has been applied to monitor RTK pharmacology, quantifying the binding of single site fluorescently-labelled VEGF_165_a (Figure 3a) to full-length human VEGFR2 in living cells at 37 °C [97].

Regardless of technique used, all VEGF-A isoforms, including those directly comparing “pro-angiogenic” VEGF_165_a and “anti-angiogenic” VEGF_165_b [77,79,97], have been shown to bind to VEGFR2 with nanomolar affinities (Table 1). Relatively lower affinities were seen for VEGF_145_a and VEGF_189_a at VEGFR2 when compared to the prototypical VEGF_165_a, demonstrated by a higher K_d_ values (Table 1). However, in terms of their pharmacology, these are small differences in affinity (VEGF_165_a K_d_ 0.15 nM vs. 1.02–1.82 nM; Table 1), particularly as physiological VEGF-A concentrations are estimated within the picomolar range [98]. As all VEGF-A isoforms contain residues that interact with VEGFR2 (Figure 2A,C) and pharmacological binding studies suggest all VEGF-A isoforms bind VEGFR2 with a similar nanomolar affinity (Table 1), this illustrates that VEGF-A/VEGFR2 binding alone is insufficient to explain functional distinctions between isoforms in terms of their signalling downstream of VEGFR2.

## 4. VEGFR2 Signalling

### 4.1. VEGFR2 Activation

**Figure 4:**
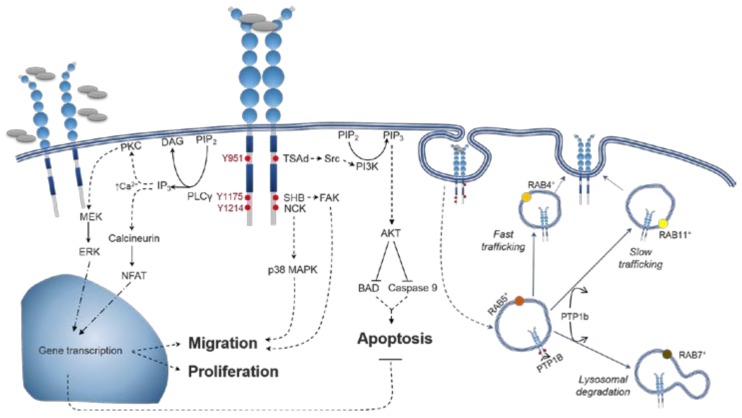
VEGFR2 signal transduction and trafficking pathways mediated by VEGF-A. Schematic representation of the signalling pathways elicited by the docking of adaptor proteins to major tyrosine phosphorylation sites. Phosphorylation of Y951 residue leads to the recruitment of TSAd which in turns binds and activates Src. Substrates for Src include molecules related to cell adhesion, vascular permeability, and cell survival (via PI3K/AKT pathway activation). pY1175 mobilises SHB, which in turn activates FAK (cell attachment and migration). SHB is also one of the Src substrates that are involved in the activation of PI3K/AKT. Moreover, pY1175 residues recruit PLCγ, triggering Ca^2+^-dependent signalling, which in turn results in transcriptional control of proliferation and cell migration. Cell motility is also regulated by the recruitment of NCK to pY1214 leading to p38MAPK activation. VEGFR2 activation promotes its own internalization with signalling continuing within endosomal compartments. After being internalized to RAB5^+^ sorting endosomes, VEGFR2 can be recycled to the cell surface in RAB4^+^ (fast trafficking, persistent intracellular signalling) or Rab11^+^ (slow trafficking, PTP1b-limited intracellular signalling) endosomes. Alternatively, VEGFR2 undergoes lysosomal degradation in Rab7^+^ endosomes. PLCγ, phospholipase Cγ; PIP_2,_ phosphatidylinositol biphosphate; DAG, diacylglycerol; IP_3_, inositol trisphosphate; PKC, protein kinase C; MAPK, mitogen-activated protein kinase; MEK, MAP/ERK kinase; ERK, extracellular signal–regulated kinases; NFAT, nuclear factor of activated T-cells; TSAd, T cell-specific adaptor protein; PI3K, phosphatidylinositol 3-kinases; PIP_3_, phosphatidylinositol triphosphate; BAD, Bcl-2-associated death promoter; SHB, Src homology-2 domain containing protein B; FAK, focal adhesion kinase; PTP1b, protein tyrosine phosphatase 1b. The dotted lines refer to signaling pathways that have additional elements to them (e.g., other adaptor proteins/non direct signaling routes) that have not been included due to space. The solid lines are for a direct signaling pathway. The blue arrows refer to the routes through which the receptor trafficked for either recycling or degradation.

VEGF-A isoforms have distinct signalling outcomes downstream of VEGFR2 activation [16]. Although VEGF-A isoforms have similar binding properties at VEGFR2, activation of VEGFR2 is a complex multi-step process. As well as VEGF-A binding its orthosteric ligand binding site, allosteric interactions can occur at topographically distinct regions [99]. Allosteric homotypic interactions between VEGFR2 monomers at Ig-like D4, D5 and D7 are an additional step necessary for VEGFR2 activation [84,100,101,102,103], as designed ankyrin repeat protein inhibitors (DARPins) can sterically block these interactions and allosterically inhibit VEGFR2 activation [103,104]. Ligand binding leads to a conformational twist throughout the extracellular region of VEGFR2 reorienting distinct Ig-like domains, shown by electron microscopy [100], small angle X-ray scattering [101], and the full-length crystal structure of structurally related VEGFR1 [88]. VEGF-A binding consequently leads to the rotation of transmembrane helices [105,106,107], with similar configurations induced by isoforms VEGF_165_a, VEGF_165_b and VEGF_121_a when measured using fluorescent resonance energy transfer (FRET) [107]. The intracellular region of VEGFR2 then undergoes conformational changes, formed of *N*- and *C*-lobes [108] with ATP binding to the flexible N-lobe cleft which enables receptor intrinsic kinase activity and phosphorylation of tyrosine residues in the C-lobe, notably Y1054 and Y1059 in the activation loop, Y951 in the kinase insert domain and Y1175 and Y1214, respectively [109]. Tyrosine phosphorylation creates binding sites for the recruitment of cytoplasmic adaptor proteins and initiates signalling pathways (reviewed in [37]). Signalling pathways downstream of VEGFR2 activation lead to numerous cellular fates (Figure 4). These include proliferation via PLCγ [110] and ERK1/2 [111], focal adhesion kinase (FAK)-mediated cell migration [112] and cell survival through phosphatidylinositol-4,5-bisphosphate 3-kinase (PI3K)/ protein kinase B (AKT) [113] (Figure 4). VEGFR2 signalling also leads to vascular permeability through FAK recruitment [114,115], p38 MAPK-mediated actin cytoskeleton reorganisation [116] and eNOS activation [117,118]. These signalling pathways undergo extensive cross-talk (reviewed in [42]). VEGFR2 is also dephosphorylated by protein phosphatase 1b (PTP1b) localised to the endoplasmic reticulum [119,120] (Figure 4), highlighting the importance of spatiotemporal trafficking on VEGFR2 activation.

### 4.2. Distinctions between VEGF-A Isoform Signalling

**Figure 5:**
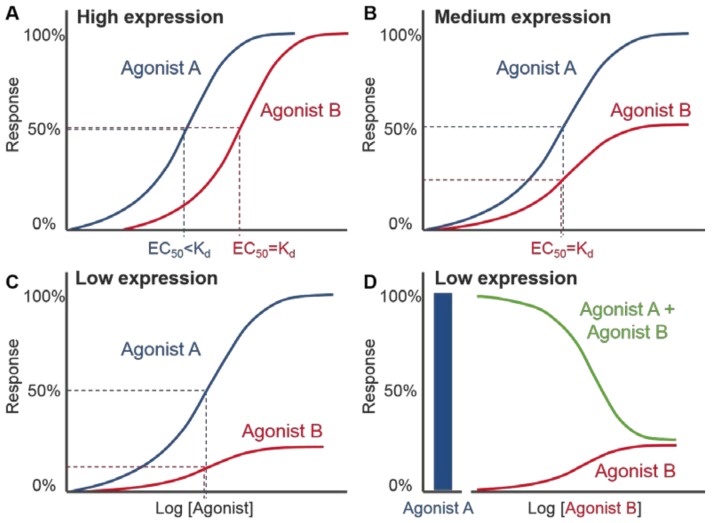
Comparison of full and partial agonists with different levels of receptor expression. (**A**) Agonist-concentration response curve in a system with high receptor expression showing two agonists A and B, which have the same dissociation constant (K_d_), produce the same maximal response and appear as full agonists. The curve for agonist A is shifted to the left (relative to agonist B) due to its higher efficacy than agonist B and ability to produce a maximum response by only occupying a small fraction of the available receptors. Agonist B has lower efficacy than agonist A and requires a higher concentration (equal to its K_d_ value) to evoke 50% maximal response. In systems with medium receptor expression (**B**) or low receptor expression (**C**), agonist B induces a lower maximal response than agonist A and can therefore be described as a partial agonist. (**D**) When the system with low receptor expression is co-stimulated with a fixed concentration of full agonist A and increasing concentrations of agonist B (green line), the partial agonist B can effectively antagonize the response to agonist A. This is because receptors occupied initially by agonist A are replaced with a lower efficacy agonist B that is only able to produce a small agonist response. The split x axis shows both the response to the fixed concentration of agonist A only (left, blue bar) and increasing log concentrations of agonist B.

Functional comparisons of the extent to which VEGF-A isoforms can drive distinct VEGFR2 signalling responses has largely come from phenotypic observations, such as changes in endothelial cell proliferation, or relative levels of phosphorylated VEGFR2/downstream signalling proteins in endothelial cells using Western blots. Despite the importance of VEGF-A concentration on both VEGFR2 binding and signalling (Figure 4), quantifying physiological VEGF-A concentrations is problematic due to the need to consider distinctions between circulating and extracellular VEGF-A, tissue-specific variation, as well as specific VEGF-A isoform concentrations sequestered in the ECM (Section 5.2). Computational modelling of systems pharmacology have predicted a relative expression of VEGF_165_ > VEGF_189_ > VEGF_121_ with total tissue, extracellular or plasma VEGF concentrations below 30 pM [98]. Functional in vitro and in vivo pharmacological experiments have typically quantified responses to agonist stimulation by the relative maximal responses induced, which can give an indication of efficacy, as well as their potency, defined as the concentration of ligand needed to produce an 50% activation/inhibition of the maximal effect (EC_50_/IC_50_) inferred from concentration-response curves [90] (Figure 5). Pharmacological investigations of VEGF-A signalling have largely been performed using fixed concentrations of ligand as opposed to full concentration response courses, making it difficult to make direct comparisons of the relative activity of isoforms across different signalling pathways and in different cellular backgrounds. This is due to the nature of agonism requiring knowledge of both affinity and efficacy, as it is not just the affinity of a ligand for its cognate receptor that governs the extent of the signalling response observed. Ligands of equal affinity can produce different maximal responses in functional assays [121,122]. They can also produce different EC_50_ values if the maximal responses are the same. This is due to the non-linear relationship that exists between receptor occupancy (i.e., the proportion of receptors that are ligand bound) and the final functional responses observed in many cells/tissues as a consequence of signal amplification [121,122]. High efficacy agonists can induce a maximum functional response even at low levels of receptor occupancy (e.g., 20%). Here, a large degree of receptor reserve is seen (i.e., EC_50_ is much lower the K_d_), meaning a high percentage of receptors (e.g., 80%) are not required to produce a maximal response. In contrast, a partial agonist cannot produce a maximal response even when receptor occupancy is 100% where receptor reserve is non-existent (e.g., no “spare” receptors are present). The extent of receptor reserve differs between agonists at the same receptor and between different cell types due to changes in receptor expression level (Figure 5), different agonist efficacies, as well as the coupling efficiency of receptors to signalling partners and the strength of signal amplification. As a result, a ligand that is a partial agonist in a signalling pathway/tissue with low receptor expression (Figure 5B,C) may exhibit full agonism when there is a higher degree of receptor reserve (Figure 5A). A single ligand may therefore show “pluridimensional efficacy” in that it displays a range of efficacies depending on the level of receptor expression, the cell background and signalling pathway observed [123].

As seen from estimations of binding affinity, all VEGF-A isoforms can bind to VEGFR2 with nanomolar affinity (Table 1, Figure 3a). However in vitro and in vivo observations have suggested that stimulation with different VEGF-A isoforms can result in distinct phenotypic outcomes (Figure 3B). Differences between isoforms can therefore not be attributable to affinity alone, but may be explained by differences in their relative intrinsic efficacies and the impact of receptor expression and signalling efficiency in different cells and tissues. The starkest of these phenotypic differences is the classification of VEGF-A isoforms into “pro-angiogenic” VEGF_xxx_a or “anti-angiogenic” VEGF_xxx_b groups. VEGF_165_a is the prototypical VEGF-A isoform that has been shown to act as a full agonist for VEGFR2 driven signalling responses observed both in vivo and in vitro and is therefore typically used as a reference ligand for investigating other VEGF-A isoforms [70,77,78,79,124,125,126,127,128,129,130,131,132]. Of the selective isoforms studied, VEGF_165_a has been shown to induce the highest levels of phosphorylation of VEGFR2 (Y1175 residue), AKT and ERK [132].

Since its discovery in 2002 [74], the VEGF_165_b isoform has been characterised as “anti-angiogenic” in relation to the prototypical VEGF_165_a. VEGF_165_b stimulation results in a reduced stimulation of angiogenesis in vivo when compared to VEGF_165_a [77,79], as well as lower tumour vessel density [133], endothelial cell proliferation [78,125,126,134], tubulogenesis and wound healing in vitro [134]. These data are consistent with VEGF_165_b acting as a low efficacy agonist in some signalling pathways rather than acting as an antagonist lacking any ability to activate VEGFR2 signalling. However, for responses in cells where VEGF_165_b is clearly acting as a weak partial agonist it will be able to antagonise the agonist effect of more efficacious agonists (e.g., VEGF_165_a) (Figure 5D). This is supported by biochemical observations that VEGF_165_b can induce phosphorylation of VEGFR2, albeit to a decreased level when compared to the full agonist VEGF_165_a [125,128], suggesting that VEGF_165_b can still partially activate VEGFR2. Additionally, VEGF_165_b can stimulate the production of NFAT in HEK293 cells expressing VEGFR2 alone, with a comparable potency to VEGF_165_a (EC_50_ VEGF_165_a 0.1 nM vs. VEGF_165_b 0.4 nM; Figure 3b) [97] albeit with decreased efficacy in respect to VEGF_165_a (68.1% ± 5.7 of maximal VEGF_165_a response; Figure 3B) [97]. Co-stimulation of VEGF_165_a with VEGF_165_b also leads to decreased maximal responses compared to VEGF_165_a alone both in vivo and in endothelial cells indicative of VEGF_165_b partial agonism [77,79]. Phenotypically, decreased vascular permeability has also been observed with VEGF_165_b treatment compared to VEGF_165_a measured by both transendothelial electrical resistance and following macromolecules conjugated to fluorescent FITC [128,129]. Cell-specific differences have been seen in respect to VEGF_165_b induced phosphorylation of AKT in HMVECs [77] and HCAECs [134] where it is decreased when compared to VEGF_165_a, but is comparable to VEGF_165_a in HPMECs [128]. Additionally, whilst differences in the extent of ERK phosphorylation were seen with VEGF_165_b vs. VEGF_165_a in HPMECs [128] and PAECs (expressing VEGFR2) [126], the levels of pERK were indistinguishable in HMVECs [77]. These differences in efficacy illustrate how the nature of VEGF-A agonism may be heavily influenced by the expression level or localisation of VEGFR2, but also other VEGF-A binding partners (VEGFR1, NRP1) or downstream adaptor proteins both in different physiological and pathological cell systems [133].

For the shorter freely diffusible VEGF-A isoform, VEGF_121_a (Figure 1), evidence exists of it exhibiting both partial and full agonism (in comparison to VEGF_165_a) depending on the signalling pathway observed. In both in vivo angiogenesis [79,125] and in vitro measurements of signalling (Figure 3b), VEGF_121_a appears to act as a partial agonist in comparison to VEGF_165_a. VEGF_121_a stimulation also leads to submaximal HUVEC proliferation when compared to VEGF_165_a [124,126,130,132], with both a rightward shift in potency and reduced maximal response also seen [67]. Compared to VEGF_165_a, VEGF_121_a also induced less HUVEC motility and sprouting [124], as well as partial calcium signalling responses [97,131]. Western blots performed by several independent groups have also suggested VEGF_121_a stimulation induces reduced phosphorylation of VEGFR2 directly [20,124,130], as well as ERK [126] and PLCγ [131] when compared to VEGF_165_a. In respect to NFAT production, VEGF_121_a showed comparable potency to VEGF_165_a (0.3 vs. 0.1 nM respectively; Figure 3B) but reduced efficacy (65.9% ± 8.8 of maximal VEGF_165_a response; Figure 3b) [97]. However, evidence exists that the extent of VEGF_121_a agonism is pathway dependent, for example, VEGF_121_a-induced ex vivo angiogenic sprouting has been shown as both comparable [70] and lower [20] than VEGF_165_a, with similar trends also seen in respect to vascular permeability seen as both comparable [132,135] and lower [136] than VEGF_165_a.

The ECM-bound isoforms, VEGF_145_a and VEGF_189_a, also show variations in the extent of agonism at VEGFR2 depending on the signalling pathway observed. Relative to VEGF_165_a, VEGF_145_a is less able to stimulate angiogenesis in vivo, albeit to a greater extent than observed with VEGF_121_a or VEGF_165_b stimulation [125]. VEGF_145_a-induced HUVEC proliferation and permeability was also partial relative to VEGF_165_a [132], however this same study also found VEGF_145_a-induced HUVEC migration was comparable to VEGF_165_a [132]. Reduced VEGFR2 phosphorylation has been observed in both murine endothelial cells [125] and HUVECs [132], as well as reduced ERK or AKT phosphorylation [132]. Although VEGF_189_a had similar functional activity to VEGF_165_a in terms of concentration-dependent proliferation in HUVECs and migration of BAECs [127], autocrine expression in isolation revealed distinctions in proliferation and cell survival [137]. Furthermore, in HEK293 cells lacking NRP1 expression and solely expressing VEGFR2, both VEGF_145_a and VEGF_189_a showed comparable potencies to VEGF_165_a in respect to NFAT production (Figure 3b) albeit with decreased efficacy (71–72% of maximal VEGF_165_a response; Figure 3b) [97].

Following its identification in 2014 [56], the novel isoform VEGF-Ax has also been characterised as “anti-angiogenic” due to its functional similarities to VEGF_165_b. Based on the early in vitro and in vivo evidence generated so far, VEGF-Ax has shown evidence of both full and partial agonism depending on the signalling pathway investigated. VEGF-Ax induced a decreased maximum response relative to VEGF_165_a in respect to VEGFR2 phosphorylation in HUVECs [56]. However, VEGF-Ax has also been shown to induce vascular permeability and ex vivo migration of HUVECs as well as promote BAEC proliferation to a comparable extent seen with VEGF_165_a stimulation [82]. VEGF-Ax possesses functional similarities to the VEGF_xxx_b isoforms, although questions remain over how frequently posttranslational read-through occurs to bypass the canonical stop codon and if this process occurs in all cells equally.

## 5. Molecular Mechanisms Distinguishing between VEGF-A Isoforms

VEGFR2 is subject to complex regulation by numerous mechanisms that underlie context-dependent signalling. It is therefore unsurprising that endogenous isoforms of varying lengths or sequences have distinct signalling outcomes with implications for altered expression in health and disease [16]. The spatial and temporal regulation of both VEGFR2 in terms of trafficking, and VEGF-A isoform bioavailability through ECM interactions, can influence isoform-specific signalling. Endothelial cells also express interaction partners NRP1 [138] and integrins [139], as well as other modulatory membrane and cytoplasmic proteins that may alter receptor expression or localisation, ultimately influencing downstream signalling and phenotypic outcomes that distinguish between VEGF-A isoforms.

### 5.1. Spatiotemporal Dynamics of VEGFR2 Trafficking

As demonstrated for other RTKs and G protein coupled receptors (reviewed in [140]), VEGFR2 undergoes endosomal signalling as it can signal from both the plasma membrane and intracellular compartments [42,132,141]. Activation of ERK requires VEGFR2 endocytosis [141], however the off-target effects of endocytic inhibitors on ERK activation requires careful interpretation [142]. VEGFR2 is localised at both the plasma membrane and in early endosomes due to constitutive VEGFR2 internalisation and recycling [143,144]. VEGFR2 can be internalised via clathrin-dependent [145,146] and -independent mechanisms [147,148]. Following VEGF-A stimulation, ligand-receptor complexes undergo internalisation within 15–20 min [97,149]. Ligand stimulation also triggers VEGFR2 recycling back to the plasma membrane [150], via short loop Rab4-positive endosomes or long loop Rab11-positive endosomes [144] (Figure 4). Alternatively, ubiquitination can initiate proteolysis and trafficking of VEGFR2 for lysosomal degradation [149,151] (Figure 4). The intracellular fate of VEGFR2—recycling or degradation—regulates the duration, amplitude and specificity of the signalling response [38,152], as the presence of activated VEGFR2 in early endosomes induces maximal ERK1/2 and AKT activation while p38 MAPK signal transduction is dependent on cell surface VEGFR2 expression [38]. Subcellular VEGFR2 trafficking is also important for receptor dephosphorylation due to the intracellular localisation of the key regulator protein phosphatase 1b (PTP1b) [119,120]. Crucially, some evidence has suggested isoform-specific trafficking of VEGFR2 [132,152], whereby VEGF_165_a induces a greater degree of ubiquitinylation than the partial agonists VEGF_121_a or VEGF_145_a [132]. Additionally, VEGFR2 internalisation can be modulated by NRP1, a co-receptor that only selected VEGF-A isoforms can interact with, rerouting VEGFR2 to long-loop recycling endosomes rather than degradative pathways [153]. VEGFR2 trafficking can also be modulated by membrane proteins VE-cadherin and ephrin B2 [145,154], however it is unknown whether these preferentially interact with specific VEGF-A isoforms. Factors modulating the spatiotemporal dynamics of VEGFR2 trafficking combined with evidence of endosomal signalling suggest a possible mechanism which may drive VEGF-A isoform specific altered signalling.

### 5.2. Spatiotemporal Dynamics of VEGF-A Bioavailability

Regulation of the bioavailability of VEGF-A isoforms following secretion may contribute to their differential biological and cellular responses [16,42]. VEGF-A isoform bioavailability is heavily influenced by their differing abilities to interact with the ECM (Figure 1). Tethering to the ECM creates localised concentrations of VEGF-A close to cells, which can be proteolytically released or cleaved to generate shorter more diffusible isoforms, creating VEGF-A gradients that can amplify VEGF-A signalling in times of angiogenic need [155]. Residues encoded by exon 6a, present in VEGF_145_, VEGF_189_ and VEGF_206_ (Figure 1), as well as exon 7-encoded residues, can interact with electronegative heparin through arginine residues confirmed by mutagenesis [70] and an NMR solution structure [156] (Figure 2B,C). Despite lacking exon 6a-encoded residues, VEGF_165_a binds heparin with relatively high affinity through exon 7-encoded arginine residues (K_d_ = 40–157 nM) [157] (Figure 2B,C). In addition to its role in sequestering VEGF-A isoforms, heparin can potentiate binding of VEGF_165_a to VEGFR2, but not freely diffusible VEGF_121_a as it lacks exon 6/7 [158,159,160]. Using SPR, Teran and Nugent (2015) did not observe any binding of VEGFR2 itself to heparin, leading them to postulate that VEGF_165_a bridges VEGFR2 and heparin so that they form a larger complex [160]. Functionally the presence of heparin increases VEGF_165_a, but not VEGF_121_a, mediated phosphorylation of VEGFR2 and enhanced VEGF_165_a-induced HUVEC mitogenesis in a dose-dependent manner [161]. Heparin also was shown to substantially enhance the affinity of interactions between VEGF_165_a and co-receptor NRP1 [162,163], illustrating the interplay between these modulatory factors on VEGF-A bioavailability and pharmacology.

### 5.3. Interactions with Co-Receptor Neuropilin-1 (NRP1)

VEGFR2 signalling is selectively enhanced by its co-receptor NRP1 [164,165], a multifaceted single transmembrane glycoprotein with which only some VEGF-A isoforms can bind (Figure 2b,c). NRP1 is also involved in neuronal guidance through binding structurally and functionally unrelated class 3 semaphorins at a distinct extracellular domain [163,166], however its functional role in vessel development is evident from the severe cardiovascular abnormalities exhibited in *Nrp1* knockout mice [167,168,169]. As with VEGFR2 [170], NRP1 upregulation in malignant tumours is correlated to aggressive cancer phenotypes [170,171,172]. NRP1 selectively potentiates VEGFR2-mediated endothelial cell motility and vascular permeability with minimal effect on proliferation, driving arterial vessel development in vivo [173,174,175]. Molecular mechanisms enhancing VEGFR2 signalling were further elucidated following the use of antibodies [124] or siRNA [20] that blocked NRP1 leading to reductions in VEGF_165_a-induced VEGFR2 and ERK phosphorylation, respectively, in vitro. VEGF-A binds the b1 domain of NRP1 [89,162,176] at exposed Tyr297 and Asp320 residue sidechains [89,177,178], primarily via an exon 8a-encoded arginine residue (CDKPRR; critical arginine underlined) with homologous interacting residues in tuftsin (TKPR) [176,179] and peptide ATWLPRR [180,181]. VEGF_165_a has a comparable binding affinity for NRP1 compared to VEGFR2 determined by radioligand binding (K_d_ < 3 nM) [92,159], while cell-free SPR-derived K_d_ values were ~100-fold higher [124,182]. With no direct VEGFR2/NRP1 binding interface identified, NRP1 is thought to modulate VEGFR2 through forming a multimeric complex bridged by NRP-binding VEGF-A isoforms. These dimeric VEGF-A ligands then interact with VEGFR2 and NRP1 via separate ends of the peptide [165,183] (Figure 2). HUVECs express more NRP1 than VEGFR2, quantified as 35,000 NRP1 receptors compared to 6000 VEGFR2 receptors per cell [152]. While NRP1-mediated VEGFR2 modulation is typically thought to arise from receptors expressed in the same cell, NRP1 expressed by adjacent cells can have distinct effects on VEGFR2 signalling, adding another layer of complexity [184]. NRP1 has a 44-amino acid intracellular domain which lacks catalytic activity, suggesting it may not be capable of signalling in its own right. However, its short cytoplasmic tail does contain a Serine-Glutamate-Alanine motif that interacts with PDZ domain-containing synectin [185,186,187]. NRP1 is suggested to modulate VEGFR2 trafficking or expression [153], likely via synectin-mediated myosin VI recruitment which is capable of transporting internalised receptors along actin filaments towards the cell body [188,189,190,191]. Interestingly VEGF-A isoforms show differences in respect to NRP binding. VEGF_165_b [79,126] and VEGF-Ax [56,82] cannot bind NRP1, whereas VEGF_165_a [79,89,92,124,125,126,159,182,192] and VEGF_189_a [193] containing exons 7- and 8a-encoded residues can bind NRP1. There are conflicting reports regarding whether VEGF_121_a, the shorter VEGF-A isoform containing exon 8a-encoded CDKPRR but lacking exon 7, can bind NRP1 (reviewed in [194]). While cell-free assays measured low affinity binding [89,124,126], other assays have not detected any interaction between VEGF_121_a and NRP1 [79,82,125], suggesting VEGF_121_a may be unable to bridge the multimeric NRP1/VEGFR2 complex. Interestingly despite containing both exon 8a-encoded (CDKPRR) and the exon 8b-encoded (SLTRKD), VEGF-Ax is unable to bind NRP1 [56,82], with suggestions that this may be due to the 22-amino acid extension present in VEGF-Ax sterically blocking binding [82]. The ability for heparin to synergistically enhance VEGF/VEGFR2/NRP1 complex formation [160] in conjunction with the differential abilities of VEGF-A isoforms to interact with NRP1, adds another layer of complexity to the VEGF/VEGFR2 signalling axis.

### 5.4. Heterodimer Formation with VEGFR1

Another potential mechanism that further diversifies VEGF/VEGFR2 signalling outcomes, is via the formation of heteroreceptor complexes between VEGFR2 and other VEGFR subtypes [195,196,197,198,199]. VEGFR2 mediated endothelial cell proliferation is negatively regulated by membrane-bound VEGFR1 homodimers [195,200]. There is a substantial decrease in VEGFR1 vs. VEGFR2 expression on the surface of endothelial cells [138], therefore VEGFR1 homodimers are likely to be relatively rare when compared to VEGFR1/VEGFR2 heterodimers [196]. Computational simulations suggest 10–50% VEGFR2 monomers are likely to exist as preassembled VEGFR1/VEGFR2 heterodimers during basal conditions with no increase upon ligand stimulation [196,201]. Enzyme-linked immunosorbent assays (ELISA) have identified the presence of preassembled VEGFR1/VEGFR2 complexes in highly vascularised organs in mice, including lung, kidney and liver [196]. These preassembled complexes have also been documented in primary cell lines, including bovine [202], porcine [203] and murine [204] endothelial cells. The use of a novel dimeric bivalent ligand (VEGF-E/PIGF-1) formed by a VEGF-E monomer (specific-ligand for VEGFR2) and a PIGF-1 monomer (specific-ligand for VEGFR1), allowed the selective activation of VEGFR1/VEGFR2 heterodimers [196]. Stimulation with VEGF-E/PIGF-1 ligand in HUVECs led to VEGFR2 phosphorylation, but relatively weak ERK1/2 phosphorylation and intracellular calcium mobilisation, compared to VEGF-A and VEGF-E alone. Additionally, VEGF-E/PIGF-1 stimulation promoted endothelial cell migration, sustained in vitro tube formation and vasodilation, but failed to mediate proliferation and endothelial factor production, suggesting that mediation of these processes may be bias towards VEGFR2 homodimers [196]. Moreover, these VEGFR1/VEGFR2 complexes also inhibited VEGF-A-induced prostacyclin release, and phosphorylation of VEGFR2 was greater in cells lacking VEGFR1, suggesting VEGFR1 may negatively modulate VEGFR2 activity in endothelial cells [196]. VEGFR2/VEGFR3 heterodimers have also been identified using proximity ligation assays [197,198,199], however, compared to VEGF-C, VEGF-A did not enhance VEGFR2/VEGFR3 heterodimer association [197]. The phosphorylation pattern for VEGFR3 homodimers and VEGFR2/3 heterodimers also showed differences [197,198,199], suggesting specific tyrosine residues may be exclusive substrates for VEGFR3 homodimers [197] and that VEGFR2/VEGFR3 heterodimers may show distinct signalling transduction and biological properties. Interestingly VEGF-A isoforms with higher affinity for heparin, such as VEGF_145_ and VEGF_189_, further potentiated VEGFR2/VEGFR3 heterodimer association when compared to VEGF_165_a or VEGF_121_a [198], providing evidence of isoform-specific heterodimer formation with functional consequences on signalling outcomes.

## 6. Conclusions and Future Perspectives

VEGF-A isoforms are distinct endogenous agonists for VEGFR2 that give rise to different functional outcomes despite similar binding properties at VEGFR2. Considering prototypical VEGF_165_a as a full agonist which stimulates maximal responses, several groups have provided evidence that other VEGF-A isoforms are partial agonists in stimulating a sub-maximal response relative to VEGF_165_a, including VEGF_165_b, VEGF_121_a, VEGF_145_a and VEGF-Ax. In terms of their molecular pharmacology, the potency and efficacy of signalling responses are both pathway- and context-dependent and heavily influenced by receptor expression and signalling protein coupling efficiency. Mechanisms distinguishing between VEGF-A isoforms, including ECM and NRP1 interactions, highlight the importance of considering VEGF-A/VEGFR2 signalling with spatiotemporal resolution. Applying quantitative pharmacological techniques used extensively with other cell surface receptor families, such as G protein coupled receptors, could further inform our molecular understanding of the VEGF/VEGFR signalling axis. The use of new technologies such as CRISPR/Cas9 would allow quantitative pharmacological observations to be performed in different cellular contexts at physiological expression levels. Endogenous VEGF-A isoform expression has been shown to be dependent on the tissue, disease state and splicing factors present, however fundamental questions remain concerning how these isoforms lead to nuances of signalling at a molecular level.

VEGF-A is critical in the development of a number of angiogenesis-dependent conditions, such as endometriosis [205], diabetic nephropathy [206], retinopathy [10], neuropathy [207], pulmonary fibrosis [61], neovascular eye diseases [208] and ischaemic heart disorders [209,210,211], as well as numerous cancer types as angiogenesis is a common hallmark for tumour development [6,212,213]. VEGF-A/VEGFR2-mediated signalling is targeted through neutralising circulating VEGF-A using bevacizumab (Avastin), blocking its receptors with RTK inhibitors (RTKIs) or inhibiting downstream signalling pathways [214,215,216]. Existing approaches lack isoform specificity, as the epitope of bevacizumab binds the VEGFR-binding region of VEGF-A (Figure 1 and Figure 2) that are present in all isoforms [217,218]. Recently, SRPK1 inhibitors have been developed to modulate VEGF-A isoform expression through favouring splicing towards partial agonist VEGF_165_b rather than full agonist VEGF_165_a [63,65]. Crucially, approved therapeutics used in oncology largely lack long-term efficacy due to the recurrent emergence of resistance mechanisms [219,220,221]. Anti-cancer therapeutics targeting VEGF-A/VEGFR2 are often used in combination with chemotherapy [25]; as a genotoxic therapeutic agent, chemotherapeutics may promote splicing to VEGF_111_ [50]. Numerous RTKIs also have on-target adverse effects due to the scope of VEGFR signalling pathways, such as hypertension caused by inhibiting pathways leading to vascular permeability [222] and vasoconstriction [223]. Molecular pharmacology forms the basis of drug development, therefore further elucidating the mechanisms that distinguish between VEGF-A isoform pharmacology and how these are orchestrated in health and in disease is fundamental to developing novel ways of targeting VEGF/VEGF receptors.

## Figures and Tables

**Table 1:** Binding affinities of VEGF-A isoforms determined at VEGFR2.

Isoform	Technique	Expression System	Binding Affinity *	Ref.
VEGF_165_a	Radioligand binding	Human kidney tissue in situ	0.01–0.04 nM	[91]
		HUVECs	0.17 nM	[92]
		Balb/c expressing VEGFR2	0.29 nM	[92]
		COS-1 cells expressing VEGFR2	0.34 nM	[92]
		PAE cells expressing VEGFR2	0.76 nM	[44]
		PAE cells expressing VEGFR2	0.097 nM	[93]
	SPR	VEGFR2 ligand binding domains (D2/D3)	36.7 nM	[85]
	ITC	VEGFR2 ligand binding domains (D2/D3)	18 nM	[85]
		VEGFR2 ligand binding domains (D2/D3)	170 nM	[84]
		VEGFR2 extracellular domain (D1–D7)	2670 nM	[84]
	NanoBRET	HEK293 cells expressing NanoLuc-VEGFR2	0.15 nM	[97]
VEGF_165_b	NanoBRET	HEK293 cells expressing NanoLuc-VEGFR2	0.39 nM	[97]
VEGF_121_a	ITC	VEGFR2 extracellular domain (D1–D7)	1120 nM	[84]
		VEGFR2 ligand binding domains (D2/D3)	93 nM	[84]
	NanoBRET	HEK293 cells expressing NanoLuc-VEGFR2	0.34 nM	[97]
VEGF_145_a	NanoBRET	HEK293 cells expressing NanoLuc-VEGFR2	1.82 nM	[97]
VEGF_189_a	NanoBRET	HEK293 cells expressing NanoLuc-VEGFR2	1.02 nM	[97]

* Ligand binding affinity quantified as equilibrium dissociation constant of the “hot” ligand (K_d_) or competing ligand (K_i_). Abbreviations: Bioluminescence resonance energy transfer (BRET) using NanoLuciferase (NanoBRET); isothermal titration calorimetry (ITC); surface plasmon resonance (SPR).

## Abbreviations

BRET	Bioluminescence Resonance Energy Transfer
eNOS	Endothelial Nitric Oxide Synthase
ERK	Extracellular Signal-Related Kinase
FAK	Focal Adhesion Kinase
GPCR	G Protein-Coupled Receptor
HEK	Human Embryonic Kidney cells
HMVECs	Human Microvascular Endothelial Cells
HPMECs	Human Pulmonary Microvascular Endothelial Cells
HUVECs	Human Umbilical Vein Endothelial Cells
MAPK	Mitogen-Activated Protein Kinases
NFAT	Nuclear Factor of Activated T-Cells
NRP1	Neuropilin-1
PAECs	Porcine Aortic Endothelial cells
PI3K	Phosphatidylinositol 3-Kinase
PLCγ	Phospholipase Cγ
RTK	Receptor Tyrosine Kinase
VEGF	Vascular Endothelial Growth Factor
VEGFR	Vascular Endothelial Growth Factor Receptor

## References

[1] Carmeliet P. (2005). Angiogenesis in life, disease and medicine. Nature.

[2] Takahashi T., Kalka C., Masuda H., Chen D., Silver M., Kearney M., Magner M., Isner J., Asahara T. (1999). Ischemia- and cytokine-induced mobilization of bone marrow-derived endothelial progenitor cells for neovascularization. Nat. Med..

[3] Tepper O.M., Capla J.M., Galiano R.D., Ceradini D.J., Callaghan M.J., Kleinman M.E., Gurtner G.C. (2005). Adult vasculogenesis occurs through in situ recruitment, proliferation, and tubulization of circulating bone marrow-derived cells. Blood.

[4] Johnson K.E., Wilgus T.A. (2014). Vascular Endothelial Growth Factor and Angiogenesis in the Regulation of Cutaneous Wound Repair. Adv. Wound Care.

[5] Lai T., Vlahos N., Shih I., Zhao Y. (2014). Expression patterns of VEGF and Flk-1 in human endometrium at the various phases of the natural menstrual cycle. Hum. Reprod..

[6] Hanahan D., Weinberg R.A. (2011). Hallmarks of cancer: The next generation. Cell.

[7] Miller J.W., Le Couter J., Strauss E.C., Ferrara N. (2013). Vascular endothelial growth factor a in intraocular vascular disease. Ophthalmology.

[8] Azizi G., Boghozian R., Mirshafiey A. (2014). The potential role of angiogenic factors in rheumatoid arthritis. Int. J. Rheum. Dis..

[9] Folkman J. (1972). Angiogenesis in psoriasis: Therapeutic implications. J. Investig. Dermatol..

[10] Ved N., Hulse R.P., Bestall S.M., Donaldson L.F., Bainbridge J.W., Bates D.O. (2017). Vascular endothelial growth factor-A 165 b ameliorates outer-retinal barrier and vascular dysfunction in the diabetic retina. Clin. Sci..

[11] Alkim C., Alkim H., Koksal A.R., Boga S., Sen I. (2015). Angiogenesis in inflammatory bowel disease. Int. J. Inflam..

[12] Pallet N., Thervet E., Timsit M.O. (2014). Angiogenic response following renal ischemia reperfusion injury: New players. Prog. Urol..

[13] Shibuya M. (2013). Vascular endothelial growth factor and its receptor system: Physiological functions in angiogenesis and pathological roles in various diseases. J. Biochem..

[14] Matsumoto K., Ema M. (2014). Roles of VEGF-A signalling in development, regeneration, and tumours. J. Biochem..

[15] Folkman J. (2007). Angiogenesis: An organizing principle for drug discovery?. Nat. Rev. Drug Discov..

[16] Woolard J., Bevan H.S., Harper S.J., Bates D. (2009). Molecular diversity of VEGF-A as a regulator of its biological activity. Microcirculation.

[17] Senger D., Galli S., Dvorak A., Perruzzi C., Harvey V., Dvorak H. (1983). Tumor Cells Secrete a Vascular Permeability Factor That Promotes Accumulation of Ascites Fluid. Science.

[18] Senger D., Perruzzi C., Feder J., Dvorak H. (1986). A Highly Conserved Vascular Permeability Factor Secreted by a Variety of Human and Rodent Tumor Cell Lines. Cancer Res..

[19] Ferrara N., Henzel W. (1989). Pituitary follicular cells secrete a novel heparin-binding growth factor specific for vascular endothelial cells. Biochem. Biophys. Res. Commun..

[20] Fearnley G.W., Odell A.F., Latham A.M., Mughal N.A., Bruns A.F., Burgoyne N.J., Homer-Vanniasinkam S., Zachary I.C., Hollstein M.C., Wheatcroft S.B. (2014). VEGF-A isoforms differentially regulate ATF-2-dependent VCAM-1 gene expression and endothelial-leukocyte interactions. Mol. Biol. Cell.

[21] Ferrara N. (2004). Vascular endothelial growth factor: Basic science and clinical progress. Endocr. Rev..

[22] Ogawa S., Oku A., Sawano A., Yamaguchi S., Yazaki Y., Shibuya M. (1998). A novel type of vascular endothelial growth factor, VEGF-E (NZ-7 VEGF). J. Biol. Chem..

[23] Yamazaki Y., Matsunaga Y., Tokunaga Y., Obayashi S., Saito M., Morita T. (2009). Snake venom vascular endothelial growth factors (VEGF-Fs) exclusively vary their structures and functions among species. J. Biol. Chem..

[24] Iyer S., Acharya K.R. (2011). Tying the knot: The cystine signature and molecular-recognition processes of the vascular endothelial growth factor family of angiogenic cytokines. FEBS J..

[25] Ferrara N., Adamis A.P. (2016). Ten years of anti-vascular endothelial growth factor therapy. Nat. Rev. Drug Discov..

[26] Muller Y.A., Heiring C., Misselwitz R., Welfle K., Welfle H. (2002). The cystine knot promotes folding and not thermodynamic stability in vascular endothelial growth factor. J. Biol. Chem..

[27] Uchida K., Uchida S., Nitta K., Yumura W., Marumo F., Nihei H. (1994). Glomerular endothelial cells in culture express and secrete vascular endothelial growth factor. Am. J. Physiol..

[28] Namiki A., Brogi E., Kearney M., Kim E.A., Wu T., Couffinhal T., Varticovski L., Isner J.M. (1995). Hypoxia induces vascular endothelial growth factor in cultured human endothelial cells. J. Biol. Chem..

[29] Nissen N.N., Polverini P.J., Koch A.E., Volin M.V., Gamelli R.L., DiPietro L.A. (1998). Vascular endothelial growth factor mediates angiogenic activity during the proliferative phase of wound healing. Am. J. Pathol..

[30] Brogi E., Wu T., Namiki A. (1994). Indirect angiogenic cytokines upregulate VEGF and bFGF gene expression in vascular smooth muscle cells, whereas hypoxia upregulates VEGF expression only. Circulation.

[31] Banks R.E., Forbes M.A., Kinsey S.E., Stanley A., Ingham E., Walters C., Selby P.J. (1998). Release of the angiogenic cytokine vascular endothelial growth factor (VEGF) from platelets: Significance for VEGF measurements and cancer biology. Br. J. Cancer.

[32] Gaudry M., Brégerie O., Andrieu V., El Benna J., Pocidalo M.-A.A., Hakim J. (1997). Intracellular pool of vascular endothelial growth factor in human neutrophils. Blood.

[33] Berse B., Brown L.F., Van De Water L., Dvorak H.F., Senger D.R. (1992). Vascular permeability factor (vascular endothelial growth factor) gene is expressed differentially in normal tissues, macrophages, and tumors. Mol. Biol. Cell.

[34] Franco M., Roswall P., Cortez E., Hanahan D., Pietras K. (2011). Pericytes promote endothelial cell survival through induction of autocrine VEGF-Asignaling and Bcl-w expression. Blood.

[35] Alexander S.P.H., Fabbro D., Kelly E., Marrion N., Peters J.A., Benson H.E., Faccenda E., Pawson A.J., Sharman J.L., Southan C. (2015). The Concise Guide to pharmacology 2015/16: Catalytic receptors. Br. J. Pharmacol..

[36] Shibuya M. (2013). VEGFR and type-V RTK activation and signaling. Cold Spring Harb. Perspect. Biol..

[37] Koch S., Tugues S., Li X., Gualandi L., Claesson-Welsh L. (2011). Signal transduction by vascular endothelial growth factor receptors. Biochem. J..

[38] Smith G.A., Fearnley G.W., Tomlinson D.C., Harrison M.A., Ponnambalam S. (2015). The cellular response to vascular endothelial growth factors requires co-ordinated signal transduction, trafficking and proteolysis. Biosci. Rep..

[39] Kabrun N., Bühring H.J., Choi K., Ullrich A., Risau W., Keller G. (1997). Flk-1 expression defines a population of early embryonic hematopoietic precursors. Development.

[40] Ishida A., Murray J., Saito Y., Kanthou C., Benzakour O., Shibuya M., Wijelath E.S. (2001). Expression of vascular endothelial growth factor receptors in smooth muscle cells. J. Cell. Physiol..

[41] Witmer A.N., Dai J., Weich H.A., Vrensen G.F., Schlingemann R.O. (2002). Expression of vascular endothelial growth factor receptors 1, 2, and 3 in quiescent endothelia. J. Histochem. Cytochem..

[42] Simons M., Gordon E., Claesson-Welsh L. (2016). Mechanisms and regulation of endothelial VEGF receptor signalling. Nat. Rev. Mol. Cell Biol..

[43] Meyer R.D., Mohammadi M., Rahimi N. (2006). A single amino acid substitution in the activation loop defines the decoy characteristic of VEGFR-1/FLT-1. J. Biol. Chem..

[44] Waltenberger J., Claesson-Welsh L., Siegbahn A., Shibuya M., Heldin C. (1994). Different Signal-Transduction Properties of Kdr and Flt1, 2 Receptors for Vascular Endothelial Growth-Factor. J. Biol. Chem..

[45] Li Y.L., Zhao H., Ren X.-B., Li Y.L., Zhao H., Ren X.B. (2016). Relationship of VEGF/VEGFR with immune and cancer cells: Staggering or forward?. Cancer Biol. Med..

[46] Sawano A., Iwai S., Sakurai Y., Ito M., Shitara K., Nakahata T., Shibuya M. (2001). Flt-1, vascular endothelial growth factor receptor 1, is a novel cell surface marker for the lineage of monocyte-macrophages in humans. Blood.

[47] Cao Y. (2009). Positive and Negative Modulation of Angiogenesis by VEGFR1 Ligands. Sci. Signal..

[48] Liu Y. (1995). Hypoxia Regulates Vascular Endothelial Growth Factor Gene Expression in Endothelial Cells. Circ. Res..

[49] Forsythe J.O.A., Jiang B., Iyer N.V., Agani F., Leung S.W. (1996). Activation of vascular endothelial growth factor gene transcription by hypoxia-inducible factor Activation of Vascular Endothelial Growth Factor Gene Transcription by Hypoxia-Inducible Factor 1. Mol. Cell. Biol..

[50] Mineur P., Colige A.C., Deroanne C.F., Dubail J., Kesteloot F., Habraken Y., Noël A., Vöö S., Waltenberger J., Lapière C.M. (2007). Newly identified biologically active and proteolysis-resistant VEGF-A isoform VEGF111 is induced by genotoxic agents. J. Cell Biol..

[51] Nowak D.G., Woolard J., Amin E.M., Konopatskaya O., Saleem M.A., Churchill A.J., Ladomery M.R., Harper S.J., Bates D.O. (2008). Expression of pro- and anti-angiogenic isoforms of VEGF is differentially regulated by splicing and growth factors. J. Cell Sci..

[52] Venables J.P. (2006). Unbalanced alternative splicing and its significance in cancer. BioEssays.

[53] Tischer E., Mitchell R., Hartman T., Silva M., Gospodarowicz D., Fiddes J.C., Abraham J.A. (1991). The Human Gene for Vascular Endothelial Growth-Factor. Multiple Protein Forms Are Encoded Through Alternative Exon Splicing. J. Biol. Chem..

[54] Guyot M., Pages G. (2015). VEGF Splicing and the Role of VEGF Splice Variants: From Physiological-Pathological Conditions to Specific Pre-mRNA Splicing.. Methods in Molecular Biology.

[55] Gu F., Li X., Kong J., Pan B., Sun M., Zheng L., Yao Y. (2013). VEGF111b, a new member of VEGFxxxb isoforms and induced by mitomycin C, inhibits angiogenesis. Biochem. Biophys. Res. Commun..

[56] Eswarappa S.M., Potdar A.A., Koch W.J., Fan Y., Vasu K., Lindner D., Willard B., Graham L.M., Dicorleto P.E., Fox P.L. (2014). Programmed translational readthrough generates antiangiogenic VEGF-Ax. Cell.

[57] Pritchard-Jones R.O., Dunn D.B.A., Qiu Y., Varey A.H.R., Orlando A., Rigby H., Harper S.J., Bates D.O. (2007). Expression of VEGFxxxb, the inhibitory isoforms of VEGF, in malignant melanoma. Br. J. Cancer.

[58] Bates D.O., Mavrou A., Qiu Y., Carter J.G., Hamdollah-Zadeh M., Barratt S., Gammons M.V., Millar A.B., Salmon A.H.J., Oltean S. (2013). Detection of VEGF-Axxxb Isoforms in Human Tissues. PLoS ONE.

[59] Dehghanian F., Hojati Z. (2014). Comparative insight into expression of recombinant human VEGF111b, a newly identified anti-angiogenic isoform, in eukaryotic cell lines. Gene.

[60] Ye X., Abou-Rayyah Y., Bischoff J., Ritchie A., Sebire N.J., Watts P., Churchill A.J., Bates D.O. (2016). Altered ratios of pro- and anti-angiogenic VEGF-A variants and pericyte expression of DLL4 disrupt vascular maturation in infantile haemangioma. J. Pathol..

[61] Barratt S.L., Blythe T., Jarrett C., Ourradi K., Shelley-Fraser G., Day M.J., Qiu Y., Harper S., Maher T.M., Oltean S. (2017). Differential Expression of VEGF-A xxx Isoforms Is Critical for Development of Pulmonary Fibrosis. Am. J. Respir. Crit. Care Med..

[62] Lambert C.A., Garbacki N., Colige A.C. (2017). Chemotherapy induces alternative transcription and splicing: Facts and hopes for cancer treatment. Int. J. Biochem. Cell Biol..

[63] Oltean S., Gammons M., Hulse R., Hamdollah-Zadeh M., Mavrou A., Donaldson L., Salmon A.H., Harper S.J., Ladomery M.R., Bates D. (2012). SRPK1 inhibition in vivo: Modulation of VEGF splicing and potential treatment for multiple diseases. Biochem. Soc. Trans..

[64] Stevens M., Oltean S. (2018). Modulation of VEGF-A Alternative Splicing as a Novel Treatment in Chronic Kidney Disease. Genes (Basel).

[65] Batson J., Toop H.D., Redondo C., Babaei-Jadidi R., Chaikuad A., Wearmouth S.F., Gibbons B., Allen C., Tallant C., Zhang J. (2017). Development of Potent, Selective SRPK1 Inhibitors as Potential Topical Therapeutics for Neovascular Eye Disease. ACS Chem. Biol..

[66] Gammons M.V., Lucas R., Dean R., Coupland S.E., Oltean S., Bates D.O. (2014). Targeting SRPK1 to control VEGF-mediated tumour angiogenesis in metastatic melanoma. Br. J. Cancer.

[67] Keyt B.A., Berleau L.T. (1996). The Carboxyl-terminal Domain(111–165) of Vascular Endothelial Growth Factor Is Critical for Its Mitogenic Potency. J. Biol. Chem..

[68] Holmes D.I.R., Zachary I.C. (2008). Vascular endothelial growth factor regulates Stanniocalcin-1 expression via Neuropilin-1-dependent regulation of KDR and synergism with fibroblast growth Factor-2. Cell Signal..

[69] Fairbrother W.J., Champe M.A., Christinger H.W., Keyt B.A., Starovasnik M.A. (1998). Solution structure of the heparin-binding domain of vascular endothelial growth factor. Structure.

[70] Krilleke D., DeErkenez A., Schubert W., Giri I., Robinson G.S., Ng Y.S., Shima D.T. (2007). Molecular mapping and functional characterization of the VEGF164 heparin-binding domain. J. Biol. Chem..

[71] Lee T.Y., Folkman J., Javaherian K. (2010). HSPG-Binding peptide corresponding to the exon 6a-encoded domain of VEGF inhibits tumor growth by blocking angiogenesis in Murine model. PLoS ONE.

[72] Houck K., Leung D.W., Rowland A.M., Winer J., Ferrara N. (1992). Dual regulation of vascular endothelial growth factor bioavailability by genetic and proteolytic mechanisms. J. Biol. Chem..

[73] Houck K.A., Ferrara N., Winer J., Cachianes G., Li B., Leung D.W. (1991). The Vascular Endothelial Growth Factor Family: Identification of a Fourth Molecular Species and Characterization of Alternative Splicing of RNA. Mol. Endocrinol..

[74] Bates D., Cui T.G., Doughty J.M., Winkler M., Sugiono M., Shields J.D., Peat D., Gillatt D., Harper S.J. (2002). VEGF165b, an inhibitory splice variant of vascular endothelial growth factor, is down-regulated in renal cell carcinoma. Cancer Res..

[75] Ladomery M.R., Harper S.J., Bates D.O. (2007). Alternative splicing in angiogenesis: The vascular endothelial growth factor paradigm. Cancer Lett..

[76] Olsson A.K., Dimberg A., Kreuger J., Claesson-Welsh L. (2006). VEGF receptor signalling–in control of vascular function. Nat. Rev. Mol. Cell Biol..

[77] Woolard J., Wang W., Bevan H.S., Qiu Y., Morbidelli L., Pritchard-Jones R.O., Cui T., Sugiono M., Waine E., Perrin R. (2004). VEGF 165b, an Inhibitory Vascular Endothelial Growth Factor Splice Variant: Mechanism of Action, In vivo Effect On Angiogenesis and Endogenous Protein Expression. Cancer Res..

[78] Catena R., Larzabal L., Larrayoz M., Molina E., Hermida J., Agorreta J., Montes R., Pio R., Montuenga L.M., Calvo A. (2010). VEGF121b and VEGF165b are weakly angiogenic isoforms of VEGF-A. Mol. Cancer.

[79] Cébe Suarez S., Pieren M., Cariolato L., Arn S., Hoffman U., Bogucki A., Manlius C., Wood J., Ballmer-Hofer K. (2006). A VEGF-A splice variant defective for heparan sulfate and neuropilin-1 binding shows attenuated signaling through VEGFR-2. Cell. Mol. Life Sci..

[80] Harris S., Craze M., Newton J., Fisher M., Shima D.T., Tozer G.M., Kanthou C. (2012). Do anti-angiogenic VEGF (VEGFxxxb) isoforms exist? a Cautionary Tale. PLoS ONE.

[81] Bridgett S., Dellett M., Simpson D.A. (2017). RNA-Sequencing data supports the existence of novel VEGFA splicing events but not of VEGFAxxxb isoforms. Sci. Rep..

[82] Xin H., Zhong C., Nudleman E., Ferrara N. (2016). Evidence for Pro-angiogenic Functions of VEGF-Ax. Cell.

[83] Roskoski R. (2008). VEGF receptor protein-tyrosine kinases: Structure and regulation. Biochem. Biophys. Res. Commun..

[84] Brozzo M.S., Bjelic S., Kisko K., Schleier T., Leppánen V.-M., Alitalo K., Winkler F.K., Ballmer-Hofer K. (2011). Thermodynamic and structural description of allosterically regulated VEGF receptor 2 dimerization. Blood.

[85] Leppanen V.M., Prota A.E., Jeltsch M., Anisimov A., Kalkkinen N., Strandin T., Lankinen H., Goldman A., Ballmer-Hofer K., Alitalo K. (2010). Structural determinants of growth factor binding and specificity by VEGF receptor 2. Proc. Natl. Acad. Sci. USA.

[86] Wiesmann C., Fuh G., Christinger H.W., Eigenbrot C., Wells J.A., de Vos A.M. (1997). Crystal Structure at 1.7 Å Resolution of VEGF in Complex with Domain 2 of the Flt-1 Receptor. Cell.

[87] Starovasnik M.A., Christinger H.W., Wiesmann C., Champe M.A., de Vos A.M., Skelton N.J. (1999). Solution structure of the VEGF-binding domain of Flt-1: Comparison of its free and bound states. J. Mol. Biol..

[88] Markovic-Mueller S., Stuttfeld E., Asthana M., Weinert T., Bliven S., Goldie K.N., Kisko K., Capitani G., Ballmer-Hofer K. (2017). Structure of the Full-length VEGFR-1 Extracellular Domain in Complex with VEGF-A. Structure.

[89] Parker M.W., Xu P., Li X., Vander Kooi C.W. (2012). Structural basis for selective vascular endothelial growth factor-A (VEGF-A) binding to neuropilin-1. J. Biol. Chem..

[90] Neubig R.R., Spedding M., Kenakin T., Christopoulos A. (2003). International Union of Pharmacology Committee on Receptor Nomenclature and Drug Classification. XXXVIII. Update on terms and symbols in quantitative pharmacology. Pharmacol. Rev..

[91] Simon M., Rockl W., Hornig C., Grone E., Theis H., Weich H., Fuchs E., Yayon A., Grone H. (1998). Receptors of Vascular Endothelial Growth Factor/Vascular Permeability Factor (VEGF/VPF) in Fetal and Adult Human Kidney : Localization and [125I] VEGF Binding. J. Am. Soc. Nephrol..

[92] Whitaker G.B., Limberg B.J., Rosenbaum J.S. (2001). Vascular Endothelial Growth Factor Receptor-2 and Neuropilin-1 Form a Receptor Complex that is Responsible for the Differential Signaling Potency of VEGF165 and VEGF121. J. Biol. Chem..

[93] Gille H., Kowalski J., Li B., LeCouter J., Moffat B., Zioncheck T.F., Pelletier N., Ferrara N. (2001). Analysis of biological effects and signaling properties of Flt-1 (VEGFR-1) and KDR (VEGFR-2): A reassessment using novel receptor-specific vascular endothelial growth factor mutants. J. Biol. Chem..

[94] Nieminen T., Toivanen P.I., Rintanen N., Heikura T., Jauhiainen S., Airenne K.J., Alitalo K., Marjomäki V., Ylä-Herttuala S. (2014). The impact of the receptor binding profiles of the vascular endothelial growth factors on their angiogenic features. Biochim. Biophys. Acta.

[95] Stoddart L., Johnstone E.K.M., Wheal A.J., Goulding J., Robers M.B., Machleidt T., Wood K.V., Hill S.J., Pfleger K.D.G. (2015). Application of BRET to monitor ligand binding to GPCRs. Nat. Methods.

[96] Stoddart L.A., Kilpatrick L.E., Hill S.J. (2017). NanoBRET Approaches to Study Ligand Binding to GPCRs and RTKs. Trends Pharmacol. Sci..

[97] Kilpatrick L.E., Friedman-Ohana R., Alcobia D., Riching K., Peach C.J., Wheal A., Briddon S., Robers M., Zimmerman K., Machleidt T. (2017). Real-time analysis of the binding of fluorescent VEGF165a to VEGFR2 in living cells: Effect of receptor tyrosine kinase inhibitors and fate of internalized agonist-receptor complexes. Biochem. Pharmacol..

[98] Clegg L.E., Mac Gabhann F. (2017). A computational analysis of in vivo VEGFR activation by multiple co-expressed ligands. PLOS Comput. Biol..

[99] De Smet F., Christopoulos A., Carmeliet P. (2014). Allosteric targeting of receptor tyrosine kinases. Nat. Biotechnol..

[100] Ruch C., Skiniotis G., Steinmetz M.O., Walz T., Ballmer-Hofer K. (2007). Structure of a VEGF–VEGF receptor complex determined by electron microscopy. Nat. Struct. Mol. Biol..

[101] Kisko K., Brozzo M.S., Missimer J., Schleier T., Menzel A., Leppänen V.-M., Alitalo K., Walzthoeni T., Aebersold R., Ballmer-Hofer K. (2011). Structural analysis of vascular endothelial growth factor receptor-2/ligand complexes by small-angle X-ray solution scattering. FASEB J..

[102] Yang Y., Xie P., Opatowsky Y., Schlessinger J. (2010). Direct contacts between extracellular membrane-proximal domains are required for VEGF receptor activation and cell signaling. Proc. Natl. Acad. Sci. USA.

[103] Hyde C.A.C., Giese A., Stuttfeld E., Abram Saliba J., Villemagne D., Schleier T., Binz H.K., Ballmer-Hofer K. (2012). Targeting extracellular domains D4 and D7 of vascular endothelial growth factor receptor 2 reveals allosteric receptor regulatory sites. Mol. Cell. Biol..

[104] Thieltges K.M., Avramovic D., Piscitelli C.L., Markovic-Mueller S., Binz H.K., Ballmer-Hofer K. (2018). Characterization of a drug-targetable allosteric site regulating vascular endothelial growth factor signaling. Angiogenesis.

[105] Dosch D.D., Ballmer-Hofer K. (2010). Transmembrane domain-mediated orientation of receptor monomers in active VEGFR-2 dimers. FASEB J..

[106] Manni S., Mineev K.S., Usmanova D., Lyukmanova E.N., Shulepko M.A., Kirpichnikov M.P., Winter J., Matkovic M., Deupi X., Arseniev A.S. (2014). Structural and functional characterization of alternative transmembrane domain conformations in VEGF receptor 2 activation. Structure.

[107] Sarabipour S., Ballmer-Hofer K., Hristova K. (2016). VEGFR-2 conformational switch in response to ligand binding. Elife.

[108] McTigue M.A., Wickersham J.A., Pinko C., Showalter R.E., Parast C.V., Tempczyk-Russell A., Gehring M.R., Mroczkowski B., Kan C.-C., Villafranca J.E. (1999). Crystal structure of the kinase domain of human vascular endothelial growth factor receptor 2: A key enzyme in angiogenesis. Structure.

[109] Manni S., Kisko K., Schleier T., Missimer J., Ballmer-Hofer K. (2014). Functional and structural characterization of the kinase insert and the carboxy terminal domain in VEGF receptor 2 activation. FASEB J..

[110] Takahashi T., Yamaguchi S., Chida K., Shibuya M. (2001). A single autophosphorylation site on KDR/Flk-1 is essential for VEGF-A-dependent activation of PLC-g and DNA synthesis in vascular endothelial cells. EMBO J..

[111] Takahashi T., Ueno H., Shibuya M. (1999). VEGF activates protein kinase C-dependent, but Ras-independent Raf-MEK-MAP kinase pathway for DNA synthesis in primary endothelial cells. Oncogene.

[112] Abu-Ghazaleh R., Kabir J., Jia H., Lobo M., Zachary I. (2001). Src mediates stimulation by vascular endothelial growth factor of the phosphorylation of focal adhesion kinase at tyrosine 861, and migration and anti-apoptosis in endothelial cells. Biochem. J..

[113] Gerber H.P., McMurtrey A., Kowalski J., Yan M., Keyt B.A., Dixit V., Ferrara N. (1998). Vascular Endothelial Growth Factor Regulates Endothelial Cell Survival through the Phosphatidylinositol 3’-Kinase/Akt Signal transduction pathway. Requirement for Flk-1/KDR activation. J. Biol. Chem..

[114] Holmqvist K., Cross M.J., Rolny C., Hägerkvist R., Rahimi N., Matsumoto T., Claesson-Welsh L., Welsh M. (2004). The adaptor protein Shb binds to tyrosine 1175 in vascular endothelial growth factor (VEGF) receptor-2 and regulates VEGF-dependent cellular migration. J. Biol. Chem..

[115] Chen X.L., Nam J.O., Jean C., Lawson C., Walsh C.T., Goka E., Lim S.T., Tomar A., Tancioni I., Uryu S. (2012). VEGF-Induced Vascular Permeability Is Mediated by FAK. Dev. Cell.

[116] McMullen M.E., Bryant P.W., Glembotski C.C., Vincent P.A., Pumiglia K.M. (2005). Activation of p38 has opposing effects on the proliferation and migration of endothelial cells. J. Biol. Chem..

[117] Lee M.Y., Luciano A.K., Ackah E., Rodriguez-Vita J., Bancroft T., Eichmann A., Simons M., Kyriakides T.R., Morales-Ruiz M., Sessa W.C. (2014). Endothelial Akt1 mediates angiogenesis by phosphorylating multiple angiogenic substrates. Proc. Natl. Acad. Sci. USA.

[118] Kang Z., Zhu H., Jiang W., Zhang S. (2013). Protocatechuic Acid Induces Angiogenesis through PI3K-Akt-eNOS-VEGF Signalling Pathway. Basic Clin. Pharmacol. Toxicol..

[119] Lanahan A.A., Lech D., Dubrac A., Zhang J., Zhuang Z.W., Eichmann A., Simons M. (2014). PTP1b is a physiologic regulator of vascular endothelial growth factor signaling in endothelial cells. Circulation.

[120] Haj F.G., Verveer P.J., Squire A., Neel B.G., Bastiaens P.I.H. (2002). Imaging Sites of Receptor Dephosphorylation by PTP1B on the Surface of the Endoplasmic Reticulum. Science.

[121] Stephenson R.P. (1956). A Modification of Receptor Theory. Br. J. Pharmacol. Chemother..

[122] Kenakin T. (2013). New concepts in pharmacological efficacy at 7TM receptors. Br. J. Pharmacol..

[123] Galandrin S., Bouvier M. (2006). Distinct Signaling Profiles of beta1 and beta2 Adrenergic Receptor Ligands toward Adenylyl Cyclase and Mitogen-Activated Protein Kinase Reveals the Pluridimensionality of Efficacy. Mol. Pharmacol..

[124] Pan Q., Chathery Y., Wu Y., Rathore N., Tong R.K., Peale F., Bagri A., Tessier-Lavigne M., Koch A.W., Watts R.J. (2007). Neuropilin-1 binds to VEGF121 and regulates endothelial cell migration and sprouting. J. Biol. Chem..

[125] Kawamura H., Li X., Harper S.J., Bates D., Claesson-Welsh L. (2008). Vascular Endothelial Growth Factor (VEGF)-A165b Is A Weak In vitro Agonist for VEGF Receptor-2 Due to Lack of Coreceptor Binding and Deficient Regulation of Kinase Activity. Cancer Res..

[126] Delcombel R., Janssen L., Vassy R., Gammons M., Haddad O., Richard B., Letourneur D., Bates D., Hendricks C., Waltenberger J. (2013). New prospects in the roles of the C-terminal domains of VEGF-A and their cooperation for ligand binding, cellular signaling and vessels formation. Angiogenesis.

[127] Hervé M.A., Buteau-Lozano H., Mourah S., Calvo F., Perrot-Applanat M. (2005). VEGF189 stimulates endothelial cells proliferation and migration in vitro and up-regulates the expression of Flk-1/KDR mRNA. Exp. Cell Res..

[128] Ourradi K., Blythe T., Jarrett C., Barratt S.L., Welsh G.I., Millar A.B. (2017). VEGF isoforms have differential effects on permeability of human pulmonary microvascular endothelial cells. Respir. Res..

[129] Pang V., Bates D.O., Leach L. (2017). Regulation of human feto-placental endothelial barrier integrity by vascular endothelial growth factors: Competitive interplay between VEGF-A165a, VEGF-A165b, PIGF and VE-cadherin. Clin. Sci..

[130] Shiying W., Boyun S., Jianye Y., Wanjun Z., Ping T., Jiang L., Hongyi H. (2017). The Different Effects of VEGFA121 and VEGFA165 on Regulating Angiogenesis Depend on Phosphorylation Sites of VEGFR2. Inflamm. Bowel Dis..

[131] Fearnley G.W., Bruns A.F., Wheatcroft S.B., Ponnambalam S. (2015). VEGF-A isoform-specific regulation of calcium ion flux, transcriptional activation and endothelial cell migration. Biol. Open.

[132] Fearnley G.W., Smith G.A., Abdul-Zani I., Yuldasheva N., Mughal N.A., Homer-Vanniasinkam S., Kearney M.T., Zachary I.C., Tomlinson D.C., Harrison M.A. (2016). VEGF-A isoforms program differential VEGFR2 signal transduction, trafficking and proteolysis. Biol. Open.

[133] Rennel E., Waine E., Guan H., Schüler Y., Leenders W., Woolard J., Sugiono M., Gillatt D., Kleinerman E., Bates D. (2008). The endogenous anti-angiogenic VEGF isoform, VEGF165b inhibits human tumour growth in mice. Br. J. Cancer.

[134] Hueso L., Rios-Navarro C., Ruiz-Sauri A., Chorro F.J., Nunez J., Sanz M.J., Bodi V., Piqueras L. (2017). Dynamics and implications of circulating anti-angiogenic VEGF-A165b isoform in patients with ST-elevation myocardial infarction. Sci. Rep..

[135] Xu D., Fuster M.M., Lawrence R., Esko J.D. (2011). Heparan sulfate regulates VEGF165- and VEGF121- mediated vascular hyperpermeability. J. Biol. Chem..

[136] Becker P.M., Waltenberger J., Yachechko R., Mirzapoiazova T., Sham J., Lee C., Elia J., Verin A. (2005). Neuropilin-1 Regulates Vascular Endothelial Growth Factor-Mediated Endothelial Permeability. Circ. Res..

[137] Yamamoto H., Rundqvist H., Branco C., Johnson R.S. (2016). Autocrine VEGF Isoforms Differentially Regulate Endothelial Cell Behavior. Front. Cell Dev. Biol..

[138] Imoukhuede P.I., Popel A.S. (2011). Quantification and cell-to-cell variation of vascular endothelial growth factor receptors. Exp. Cell Res..

[139] Baranska P., Jerczynska H., Pawlowska Z., Koziolkiewicz W., Cierniewski C.S. (2005). Expression of Integrins and Adhesive Properties of Human Endothelial Cell Line EA.hy 926. Cancer Genom. Proteom..

[140] Murphy J.E., Padilla B.E., Hasdemir B., Cottrell G.S., Bunnett N.W. (2009). Endosomes: A legitimate platform for the signaling train. Proc. Natl. Acad. Sci. USA.

[141] Gourlaouen M., Welti J.C., Vasudev N.S., Reynolds A.R. (2013). Essential Role for Endocytosis in the Growth Factor-stimulated Activation of ERK1/2 in Endothelial Cells. J. Biol. Chem..

[142] Basagiannis D., Zografou S., Galanopoulou K., Christoforidis S. (2017). Dynasore impairs VEGFR2 signalling in an endocytosis- independent manner. Sci. Rep..

[143] Jopling H.M., Howell G.J., Gamper N., Ponnambalam S. (2011). The VEGFR2 receptor tyrosine kinase undergoes constitutive endosome-to-plasma membrane recycling. Biochem. Biophys. Res. Commun..

[144] Jopling H.M., Odell A.F., Pellet-Many C., Latham A.M., Frankel P., Sivaprasadarao A., Walker J.H., Zachary I.C., Ponnambalam S. (2014). Endosome-to-Plasma Membrane Recycling of VEGFR2 Receptor Tyrosine Kinase Regulates Endothelial Function and Blood Vessel Formation. Cells.

[145] Lampugnani M.G., Orsenigo F., Gagliani M.C., Tacchetti C., Dejana E. (2006). Vascular endothelial cadherin controls VEGFR-2 internalization and signaling from intracellular compartments. J. Cell Biol..

[146] Ewan L.C., Jopling H.M., Jia H., Mittar S., Bagherzadeh A., Howell G.J., Walker J.H., Zachary I.C., Ponnambalam S. (2006). Intrinsic tyrosine kinase activity is required for vascular endothelial growth factor receptor 2 ubiquitination, sorting and degradation in endothelial cells. Traffic.

[147] Basagiannis D., Christoforidis S. (2016). Constitutive endocytosis of VEGFR2 protects the receptor against shedding. J. Biol. Chem..

[148] Basagiannis D., Zografou S., Murphy C., Fotsis T., Morbidelli L., Ziche M., Bleck C., Mercer J., Christoforidis S. (2016). VEGF induces signalling and angiogenesis by directing VEGFR2 internalisation via macropinocytosis. J. Cell Sci..

[149] Smith G.A., Fearnley G.W., Abdul-Zani I., Wheatcroft S.B., Tomlinson D.C., Harrison M.A., Ponnambalam S. (2016). VEGFR2 Trafficking, Signaling and Proteolysis is Regulated by the Ubiquitin Isopeptidase USP8. Traffic.

[150] Gampel A., Moss L., Jones M.C., Brunton V., Norman J.C., Mellor H. (2006). VEGF regulates the mobilization of VEGFR2/KDR from an intracellular endothelial storage compartment. Blood.

[151] Bruns A.F., Herbert S.P., Odell A.F., Jopling H.M., Hooper N.M., Zachary I.C., Walker J.H., Ponnambalam S. (2010). Ligand-stimulated VEGFR2 signaling is regulated by co-ordinated trafficking and proteolysis. Traffic.

[152] Clegg L.W., Mac Gabhann F. (2015). Site-Specific Phosphorylation of VEGFR2 Is Mediated by Receptor Trafficking: Insights from a Computational Model. PLOS Comput. Biol..

[153] Ballmer-Hofer K., Andersson A.E., Ratcliffe L.E., Berger P. (2011). Neuropilin-1 promotes VEGFR-2 trafficking through Rab11 vesicles thereby specifying signal output. Blood.

[154] Wang Y., Nakayama M., Pitulescu M.E., Schmidt T.S., Bochenek M.L., Sakakibara A., Adams S., Davy A., Deutsch U., Lüthi U. (2010). Ephrin-B2 controls VEGF-induced angiogenesis and lymphangiogenesis. Nature.

[155] Vempati P., Popel A.S., Mac Gabhann F. (2014). Extracellular regulation of VEGF: Isoforms, proteolysis, and vascular patterning. Cytokine Growth Factor Rev..

[156] Melissa E.S., Skelton N.J., Fairbrother W.J. (2002). Refinement of the solution structure of the heparin-binding domain of vascular endothelial growth factor using residual dipolar couplings. J. Biomol. NMR.

[157] Zhao W., McCallum S.A., Xiao Z., Zhang F., Linhardt R.J. (2012). Binding affinities of vascular endothelial growth factor (VEGF) for heparin-derived oligosaccharides. Biosci. Rep..

[158] Gitay-Goren H., Soker S., Vlodavsky I., Neufeld G. (1992). The binding of vascular endothelial growth factor to its receptors is dependent on cell surface-associated heparin-like molecules. J. Biol. Chem..

[159] Soker S., Takashima S., Miao H.Q., Neufeld G., Klagsbrun M. (1998). Neuropilin-1 Is Expressed by Endothelial and Tumor Cells as an Isoform-Specific Receptor for Vascular Endothelial Growth Factor. Cell.

[160] Teran M., Nugent M.A. (2015). Synergistic binding of vascular endothelial growth factor-a and its receptors to heparin selectively modulates complex affinity. J. Biol. Chem..

[161] Ashikari-Hada S., Habuchi H., Kariya Y., Kimata K. (2005). Heparin regulates vascular endothelial growth factor165-dependent mitogenic activity, tube formation, and its receptor phosphorylation of human endothelial cells. Comparison of the effects of heparin and modified heparins. J. Biol. Chem..

[162] Mamluk R., Gechtman Z., Kutcher M.E., Gasiunas N., Gallagher J., Klagsbrun M. (2002). Neuropilin-1 binds vascular endothelial growth factor 165, placenta growth factor-2, and heparin via its b1b2 domain. J. Biol. Chem..

[163] Gu C., Limberg B.J., Brian Whitaker G., Perman B., Leahy D.J., Rosenbaum J.S., Ginty D.D., Kolodkin A.L. (2002). Characterization of neuropilin-1 structural features that confer binding to semaphorin 3A and vascular endothelial growth factor 165. J. Biol. Chem..

[164] Djordjevic S., Driscoll P.C. (2013). Targeting VEGF signalling via the neuropilin co-receptor. Drug Discov. Today.

[165] Guo H.F., Vander Kooi C.W. (2015). Neuropilin Functions as an Essential Cell Surface Receptor. J. Biol. Chem..

[166] Appleton B., Wu P., Maloney J., Yin J.P., Liang W.-C., Stawicki S., Mortara K., Bowman K.K., Elliott J.M., Desmarais W. (2007). Structural studies of neuropilin/antibody complexes provide insights into semaphorin and VEGF binding. EMBO J..

[167] Kitsukawa T., Shimizu M., Sanbo M., Hirata T., Taniguchi M., Bekku Y., Yagi T., Fujisawa H. (1997). Neuropilin–Semaphorin III/D-Mediated Chemorepulsive Signals Play a Crucial Role in Peripheral Nerve Projection in Mice. Neuron.

[168] Kawasaki T., Kitsukawa T., Bekku Y., Matsuda Y., Sanbo M., Yagi T., Fujisawa H. (1999). A requirement for neuropilin-1 in embryonic vessel formation. Development.

[169] Gu C., Rodriguez E.R., Reimert D.V., Shu T., Fritzsch B., Richards L.J., Kolodkin A.L., Ginty D.D. (2003). Neuropilin-1 conveys semaphorin and VEGF signaling during neural and cardiovascular development. Dev. Cell.

[170] Goel H.L., Mercurio A.M. (2013). VEGF targets the tumour cell. Nat. Rev. Cancer.

[171] Jubb A.M., Strickland L.A., Liu S.D., Mak J., Schmidt M., Koeppen H. (2012). Neuropilin-1 expression in cancer and development. J. Pathol..

[172] Lee S.W., Lee J.E., Yoo C.Y., Ko M.S., Park C.S., Yang S.H. (2014). NRP-1 expression is strongly associated with the progression of pituitary adenomas. Oncol. Rep..

[173] Chittenden T.W., Claes F., Lanahan A.A., Autiero M., Palac R.T., Tkachenko E.V., Elfenbein A., Ruiz de Almodovar C., Dedkov E., Tomanek R. (2006). Selective Regulation of Arterial Branching Morphogenesis by Synectin. Dev. Cell.

[174] Fantin A., Schwarz Q., Davidson K., Normando E.M., Denti L., Ruhrberg C. (2011). The cytoplasmic domain of neuropilin 1 is dispensable for angiogenesis, but promotes the spatial separation of retinal arteries and veins. Development.

[175] Lanahan A., Zhang X., Fantin A., Zhuang Z., Rivera-Molina F., Speichinger K., Prahst C., Zhang J., Wang Y., Davis G. (2013). The neuropilin 1 cytoplasmic domain is required for VEGF-A-dependent arteriogenesis. Dev. Cell.

[176] Vander Kooi C.W., Jusino M.A., Perman B., Neau D.B., Bellamy H.D., Leahy D.J. (2007). Structural basis for ligand and heparin binding to neuropilin B domains. Proc. Natl. Acad. Sci. USA.

[177] Mota F., Fotinou C., Rhana R., Edith Chan A.W., Yelland T., Arooz M.T., O’Leary A.P., Hutton J., Frankel P., Zachary I. (2018). Architecture and Hydration of the Arginine Binding Site of Neuropilin-1. FEBS J..

[178] Herzog B., Pellet-Many C., Britton G., Hartzoulakis B., Zachary I.C. (2011). VEGF binding to NRP1 is essential for VEGF stimulation of endothelial cell migration, complex formation between NRP1 and VEGFR2, and signaling via FAK Tyr407 phosphorylation. Mol. Biol. Cell.

[179] Von Wronski M.A., Raju N., Pillai R., Bogdan N.J., Marinelli E.R., Nanjappan P., Ramalingam K., Arunachalam T., Eaton S., Linder K.E. (2006). Tuftsin binds neuropilin-1 through a sequence similar to that encoded by exon 8 of vascular endothelial growth factor. J. Biol. Chem..

[180] Starzec A., Ladam P., Vassy R., Badache S., Bouchemal N., Navaza A., du Penhoat C.H., Perret G.Y. (2007). Structure-function analysis of the antiangiogenic ATWLPPR peptide inhibiting VEGF165 binding to neuropilin-1 and molecular dynamics simulations of the ATWLPPR/neuropilin-1 complex. Peptides.

[181] Starzec A., Vassy R., Martin A., Lecouvey M., Di Benedetto M., Crépin M., Perret G.Y. (2006). Antiangiogenic and antitumor activities of peptide inhibiting the vascular endothelial growth factor binding to neuropilin-1. Life Sci..

[182] Fuh G., Garcia K.C., de Vos A.M. (2000). The interaction of neuropilin-1 with vascular endothelial growth factor and its receptor flt-1. J Biol.Chem..

[183] Parker M.W., Guo H.F., Li X., Linkugel A.D., Vander Kooi C.W. (2012). Function of members of the neuropilin family as essential pleiotropic cell surface receptors. Biochemistry.

[184] Koch S., Van Meeteren L.A., Morin E., Testini C., Weström S., Björkelund H., Le Jan S., Adler J., Berger P., Claesson-Welsh L. (2014). NRP1 Presented in trans to the endothelium arrests VEGFR2 endocytosis, preventing angiogenic signaling and tumor initiation. Dev. Cell.

[185] Cai H., Reed R.R. (1999). Cloning and characterization of neuropilin-1-interacting protein: A PSD-95/Dlg/ZO-1 domain-containing protein that interacts with the cytoplasmic domain of neuropilin-1. J. Neurosci..

[186] Wang L., Mukhopadhyay D., Xu X. (2006). C terminus of RGS-GAIP-interacting protein conveys neuropilin-1-mediated signaling during angiogenesis. FASEB J..

[187] Prahst C., Héroult M., Lanahan A.A., Uziel N., Kessler O., Shraga-Heled N., Simons M., Neufeld G., Augustin H.G. (2008). Neuropilin-1-VEGFR-2 complexing requires the PDZ-binding domain of neuropilin-1. J. Biol. Chem..

[188] Wells A.L., Lin A.W., Chen L.Q., Safer D., Cain S.M., Hasson T., Carragher B.O., Milligan R.A., Sweeney H.L. (1999). Myosin VI is an actin-based motor that moves backwards. Nature.

[189] Shang G., Brautigam C.A., Chen R., Lu D., Torres-vázquez J. (2017). Structure analyses reveal a regulated oligomerization mechanism of the PlexinD1/GIPC/myosin VI complex. Elife.

[190] Naccache S., Hasson T., Horowitz A. (2006). Binding of internalized receptors to the PDZ domain of GIPC/synectin recruits myosin VI to endocytic vesicles. PNAS.

[191] Reed B., Cefalu C., Bellaire B., Cardelli J., Louis T., Salamon J., Bloecher M., Bunn R. (2005). GLUT1CBP(TIP2/GIPC1) Interactions with GLUT1 and Myosin VI: Evidence Supporting an Adapter Function for GLUT1CBP. Mol. Biol. Cell.

[192] Parker M.W., Xu P., Guo H.F., Vander Kooi C.W. (2012). Mechanism of Selective VEGF-A Binding by Neuropilin-1 Reveals a Basis for Specific Ligand Inhibition. PLoS ONE.

[193] Tillo M., Erskine L., Cariboni A., Fantin A., Joyce A., Denti L., Ruhrberg C. (2015). VEGF189 binds NRP1 and is sufficient for VEGF/NRP1-dependent neuronal patterning in the developing brain. Development.

[194] Sarabipour S., Mac Gabhann F. (2017). VEGF-A121a binding to Neuropilins–A concept revisited. Cell Adh. Migr..

[195] Rahimi N., Dayanir V., Lashkari K. (2000). Receptor chimeras indicate that the vascular endothelial growth factor receptor-1 (VEGFR-1) modulates mitogenic activity of VEGFR-2 in endothelial cells. J. Biol. Chem..

[196] Cudmore M.J., Hewett P.W., Ahmad S., Wang K.-Q., Cai M., Al-Ani B., Fujisawa T., Ma B., Sissaoui S., Ramma W. (2012). The role of heterodimerization between VEGFR-1 and VEGFR-2 in the regulation of endothelial cell homeostasis. Nat. Commun..

[197] Dixelius J., Mäkinen T., Wirzenius M., Karkkainen M.J., Wernstedt C., Alitalo K., Claesson-Welsh L. (2003). Ligand-induced Vascular Endothelial Growth Factor Receptor-3 (VEGFR-3) Heterodimerization with VEGFR-2 in Primary Lymphatic Endothelial Cells Regulates Tyrosine Phosphorylation Sites. J. Biol. Chem..

[198] Nilsson I., Bahram F., Li X., Gualandi L., Koch S., Jarvius M., Söderberg O., Anisimov A., Kholová I., Pytowski B. (2010). VEGF receptor 2/-3 heterodimers detected in situ by proximity ligation on angiogenic sprouts. EMBO J..

[199] Coon B.G., Baeyens N., Han J., Budatha M., Ross T.D., Fang J.S., Yun S., Thomas J.-L., Schwartz M.A. (2015). Intramembrane binding of VE-cadherin to VEGFR2 and VEGFR3 assembles the endothelial mechanosensory complex. J. Cell Biol..

[200] Bussolati B., Dunk C., Grohman M., Kontos C.D., Mason J., Ahmed A. (2001). Vascular Endothelial Growth Factor Receptor-1 Modulates Vascular Endothelial Growth Factor-Mediated Angiogenesis via Nitric Oxide. Am. J. Pathol..

[201] Mac Gabhann F., Popel A.S. (2007). Dimerization of VEGF receptors and implications for signal transduction: A computational study. Biophys. Chem..

[202] Neagoe P.E., Lemieux C., Sirois M.G. (2005). Vascular endothelial growth factor (VEGF)-A165-induced prostacyclin synthesis requires the activation of VEGF receptor-1 and -2 heterodimer. J. Biol. Chem..

[203] Huang K., Andersson C., Roomans G.M., Ito N., Claesson-Welsh L. (2001). Signaling properties of VEGF receptor-1 and -2 homo- and heterodimers. Int. J. Biochem. Cell Biol..

[204] Autiero M., Waltenberger J., Communi D., Kranz A., Moons L., Lambrechts D., Kroll J., Plaisance S., de Mol M., Bono F. (2003). Role of PlGF in the intra- and intermolecular cross talk between the VEGF receptors Flt1 and Flk1. Nat. Med..

[205] Danastas K., Miller E.J., Hey-Cunningham A.J., Murphy C.R., Lindsay L.A. (2017). Expression of vascular endothelial growth factor A isoforms is dysregulated in women with endometriosis. Reprod. Fertil. Dev..

[206] Majumder S., Advani A. (2016). VEGF and the diabetic kidney: More than too much of a good thing. J. Diabetes Complic..

[207] Hulse R.P., Beazley-Long N., Ved N., Bestall S.M., Riaz H., Singhal P., Ballmer-Hofer K., Harper S.J., Bates D., Donaldson L.F. (2015). Vascular endothelial growth factor-A 165b prevents diabetic neuropathic pain and sensory neuronal degeneration. Clin. Sci..

[208] Rubio R.G., Adamis A.P. (2016). Ocular Angiogenesis: Vascular Endothelial Growth Factor and Other Factors. Dev. Ophthalmol..

[209] Shibata Y., Kikuchi R., Ishii H., Suzuki S., Harada K., Hirayama K., Suzuki A., Tatami Y., Kondo K., Murohara T. (2018). Balance between angiogenic and anti-angiogenic isoforms of VEGF-A is associated with the complexity and severity of coronary artery disease. Clin. Chim. Acta.

[210] Ganta V.C., Choi M., Kutateladze A., Annex B.H. (2016). VEGF165b Modulates Endothelial VEGFR1-STAT3 Signaling Pathway and Angiogenesis in Human and Experimental Peripheral Arterial Disease. Pediatr. Neurol..

[211] Kikuchi R., Nakamura K., Maclauchlan S., Ngo D.T. (2015). An anti-angiogenic isoform of VEGF-A contributes to impaired vascularization in peripheral artery disease. Nat. Med..

[212] Sia D., Clara A., Newell P., Villanueva A. (2014). VEGF signaling in cancer treatment. Curr. Pharm. Des..

[213] Rapisarda A., Melillo G. (2012). Role of the VEGF/VEGFR Axis in Cancer Biology and Therapy. Adv. Cancer Res..

[214] Guyot M., Hilmi C., Ambrosetti D., Merlano M., Lo Nigro C., Durivault J., Grépin R., Pagès G. (2016). Targeting the pro-angiogenic forms of VEGF or inhibiting their expression as anti-cancer strategies. Oncotarget.

[215] Amadio M., Govoni S., Pascale A. (2016). Targeting VEGF in eye neovascularization: What’s new?: A comprehensive review on current therapies and oligonucleotide-based interventions under development. Pharmacol. Res..

[216] Comunanza V., Bussolino F. (2017). Therapy for Cancer: Strategy of Combining Anti-Angiogenic and Target Therapies. Front. Cell Dev. Biol..

[217] Kim K.J., Li B., Houck K., Winer J., Ferrara N. (1992). The Vascular Endothelial Growth Factor Proteins: Identification of Biologically Relevant Regions by Neutralizing Monoclonal Antibodies. Growth Factors.

[218] Varey A.H.R., Rennel E.S., Qiu Y., Bevan H.S., Perrin R.M., Raffy S., Dixon A.R., Paraskeva C., Zaccheo O., Hassan A.B. (2008). VEGF165b, an antiangiogenic VEGF-A isoform, binds and inhibits bevacizumab treatment in experimental colorectal carcinoma: Balance of pro- and antiangiogenic VEGF-A isoforms has implications for therapy. Br. J. Cancer.

[219] Simon T., Gagliano T., Giamas G. (2017). Direct Effects of Anti-Angiogenic Therapies on Tumor Cells: VEGF Signaling. Trends Mol. Med..

[220] Yang S., Zhao J., Sun X. (2016). Resistance to anti-VEGF therapy in neovascular age-related macular degeneration : A comprehensive review. Drug Des. Dev. Ther..

[221] Bahrami B., Zhu M., Hong T., Chang A. (2016). Diabetic macular oedema: Pathophysiology, management challenges and treatment resistance. Diabetologia.

[222] Schmidinger M. (2013). Understanding and managing toxicities of vascular endothelial growth factor (VEGF) inhibitors. Eur. J. Cancer Suppl..

[223] Carter J.J., Fretwell L.V., Woolard J. (2017). Effects of 4 multitargeted receptor tyrosine kinase inhibitors on regional hemodynamics in conscious, freely moving rats. FASEB J..

